# The inter-association between face processing, intelligence, and autistic-like nonverbal communication

**DOI:** 10.1177/17470218251323388

**Published:** 2025-02-12

**Authors:** Dana L Walker, Romina Palermo, Gilles E Gignac

**Affiliations:** 1 School of Psychological Science, The University of Western Australia, Perth, WA, Australia

**Keywords:** Face recognition, emotion recognition, intelligence, CHC, trait-autism, developmental prosopagnosia

## Abstract

The degree to which face processing abilities inter-relate, and associate with general intelligence, remains a contentious issue. Furthermore, poorer face processing abilities may be a result of reduced social interest associated with higher levels of trait-autism, consistent with the social motivation theory of autism. However, the association between multiple dimensions of face processing (i.e., a general face factor) and trait-autism, specifically autistic-like nonverbal communication, has not been estimated. Consequently, we administered four face processing ability tests (assessing face detection, the perception and memory of face identity, and expression recognition), four cognitive ability tests, and the Autism Quotient to a sample of 253 general community adults. Based on latent variable modelling, we identified a general face processing ability factor (*f*), and it was positively associated with general intelligence (*g*; λ = .48). We conclude that face processing abilities may be a candidate ability within the Cattell–Horn–Carroll model of intelligence. Moreover, face memory was positively associated with *g* (β = .31). We discuss the possibility of developmental prosopagnosia, i.e., deficits in face memory, being diagnosed as a learning disability. Furthermore, autistic-like nonverbal communication was a significant, negative predictor (β = −.45) of *f*, and *g* was neither a mediator nor suppressor of the effect. Finally, the unique effect between autistic-like nonverbal communication difficulties and face processing abilities, independently of intelligence, was considered in line with the social motivation theory of autism.

## Introduction

The human face conveys important social information, and the ability to process face-related information can facilitate the effective development and maintenance of social relationships (Palermo et al., 2013; Russell et al., 2009). Dimensions of face processing ability include face detection, face perception, face memory and, expression recognition.^1^ These four processing abilities can be conceptualised as dimensions, such that performance occurs along a continuum, from severe impairment to exceptional performance, with mostly “average” or typical ability. There is evidence for a face processing ability positive manifold and possible common factor (Hildebrandt et al., 2011; Olderbak et al., 2015; Verhallen et al., 2017; Wilhelm et al., 2010); however, no research has been conducted on the inter-association between all four of these key face processing abilities.

Furthermore, the association between face processing abilities and general intelligence remains to be determined. Some researchers have contended that perceiving and recognising facial identity, as well as expression recognition, is independent from general intelligence (e.g., Bowles et al., 2009; Palermo et al., 2013; Shakeshaft & Plomin, 2015; Wilmer et al., 2014), while others have provided empirical evidence of a positive association (e.g., Connolly et al., 2019; Gignac et al., 2016; Hildebrandt et al., 2011). Based on a recent review of the empirical literature, studies that reported significant, positive effects between intelligence and the detection, perception and recognition of facial identity tended to use respectable samples (size and representativeness) and intelligence measurement (Walker et al., 2023a). Similarly, a meta-analysis between intelligence and expression recognition noted an under-reporting of reliability statistics for intelligence tests and a relatively low mean reliability for expression recognition tests (Schlegel et al., 2019). Thus, it is possible that an appreciable positive association between intelligence and face processing could be estimated when adequate methodology is implemented.

Finally, research suggests an association between trait-autism, personality traits associated with autism observed within the neurotypical populations, and face processing abilities, such as facial expression (e.g., Bothe et al., 2019; Lewis et al., 2016; Poljac et al., 2012) and face identity recognition (e.g., Davis et al., 2017; Halliday et al., 2014; Lewis et al., 2018). Typically, individuals with higher levels of trait-autism, particularly those exhibiting challenges in nonverbal communication often associated with autism, tend to show lower performance in tasks related to recognising facial expressions and facial identities.

To date, no research has investigated the shared variance between these four specific face processing abilities and trait-autism. An interesting question is whether the association between face processing abilities and trait-autism would increase when controlling for general intelligence. Such a hypothesis is plausible, considering general intelligence is likely unrelated to trait-autism (Folstein et al., 1999), but positively related to face processing abilities. Thus, a classic suppressor effect may be observed (Paulhus et al., 2004), whereby the strength of the association between face processing abilities and trait-autism may increase and provide a more accurate estimation of the true effect size.

In summary, the main purpose of this investigation was to: (1) estimate the shared variance between four key face processing abilities; (2) estimate the degree to which cognitive abilities are associated with these face processing abilities; and (3) test a model predicting face processing via the nonverbal communication dimension of trait-autism and cognitive abilities. Next, we describe each of the four face processing abilities in more detail.

### Delineating face processing abilities

Face detection is an individual’s ability to detect a face when viewing a visual scene. Faces are an important object to detect and studies suggest there may be a dedicated neurophysiological system that enables faces to be detected faster than non-face objects (Wardle et al., 2020). One of the more common measures of face detection is the Mooney Face Task (Mooney, 1957), where participants must select the image that contains a face out of three degraded black and white images (Verhallen & Mollon, 2016; for a short version, see Walker et al., 2023b). Face detection is an important first step for higher-order cognitive processes associated with face processing, such as face recognition (Wardle et al., 2020). Correspondingly, there are cases where people with prosopagnosia (i.e., profound deficits in face identity recognition) manifest substantial deficits in face detection ability, an observation thought to contribute to their impairments in higher-order face processing abilities, such as face memory (de Gelder & Stekelenburg, 2005; Garrido et al., 2008).

Face perception occurs after a face is detected and involves discriminating or individualising faces from each other. Face perception is commonly measured with the Cambridge Face Perception Test (CFPT; Duchaine et al., 2007), the Glasgow Face Matching Task (GFMT & GFMT2; Burton et al., 2010 and White et al., 2022, respectively), or the Benton Facial Recognition Test (Benton et al., 1983). In face perception tasks, the face stimuli remain visible, minimising the memory requirements and focusing more on the visual processing of faces. Development of face perception ability may be a precursor to development of face memory, a higher-order face identity ability, sharing approximately 25% of variance (McCaffery et al., 2018; Verhallen et al., 2017). Despite this, face perception is a distinct face processing skill with diverse utility. For example, professionals, such as police officers, who scan video footage to match face images of known suspects, or airport border security officers, who need to match the passport photo with the individual standing in front of them (White & Burton, 2022; White et al., 2021).

Face memory, commonly referred to as face recognition, is the ability to view a face, encode it to memory, and recall that face at a later time (Dalrymple & Palermo, 2016). The Cambridge Face Memory Test (CFMT; Duchaine & Nakayama, 2006) is a widely used measure of face memory. In this task, participants must memorise six unfamiliar male faces (memorisation phase), and shortly after, they are presented with three face images and asked to identify the face they had previously memorised (test phase). The short timeframe between the memorisation phrase and test phase of the CFMT indicates that this is a measure of short-term face memory, rather than long-term face memory.^2^ This measure is valid, reliable, and sensitive to individual differences, and therefore, is commonly assessed to identify deficits in face memory, such as prosopagnosia (Duchaine & Nakayama, 2006), and measure individual differences in face memory within the typical population (e.g., Bowles et al., 2009).

Face memory is important in social situations, as people often need to accurately recall an individual’s identity and act appropriately for that social situation. Face memory ability exists on a continuum, from the most severe form of face blindness known as prosopagnosia (Duchaine & Nakayama, 2006) to those with incredibly accurate face memory ability known as super recognisers (Russell et al., 2009), with a majority of people somewhere in-between. Due to their inability to recognise faces, individuals with prosopagnosia tend to report more frequent and severe social difficulties, leading to negative outcomes, such as (but not restricted to) limited social circles, a high dependency on others in social settings, difficulty with relationships, low self-confidence, and fewer employment opportunities (Murray et al., 2018; Yardley et al., 2008). Therefore, face memory is an important skill for socialisation and deficits in this ability have a substantial negative impact on different aspects of an individual’s life.

The final face processing ability we investigated is face expression recognition ability, i.e., the ability to accurately identify facial expressions, a process that can help inform about another person’s emotional state. Research suggests that successful interpersonal interactions are often highly dependent on how well someone can identify facial expressions related to emotion and appropriately respond (Fox & Zougkou, 2011). A psychometrically established measure of face expression recognition ability is the Emotion Labelling Task (Palermo et al., 2018). This task involves viewing an expressive face and selecting the appropriate emotion label. There are six possible emotional labels in this task: anger, disgust, fear, happy, sad, and surprised. Across 144 trials (or items), participants view a face expressing an emotion and are asked to identify the emotion expressed using one of the six emotion labels. This task is sensitive to individual variation and is a common measure in individual difference research for expression recognition.

### Positive manifold for intelligence and the Cattell–Horn–Carroll model

A positive manifold is present when positive correlations are observed between measures of cognitive ability. Such correlations tend to yield a common (general) factor of intelligence (Jensen, 1998; Spearman, 1904). Today, a popular, comprehensive model that describes the structure of human cognitive abilities is the Cattell–Horn–Carroll (CHC) model (Flanagan & Dixon, 2013; McGrew, 2009). As described by Newton and McGrew (2010), the model consists of three stratums: a general intelligence factor (*g*; Stratum III), nine broad abilities (Stratum II) and over 100 narrow abilities (Stratum I). Examples of some of the Stratum II abilities include crystallised intelligence (*Gc*), short-term memory (*Gsm*), processing speed (*Gs*), and visuospatial ability (*Gv*). Stratum I and Stratum II abilities are positively associated with *g*, to varying degrees. These positive intercorrelations between Stratum II abilities allow for the estimation of *g*; however, these abilities are still, to some degree, unique and distinguishable from each other (Flanagan & Dixon, 2013).

The original formulation of the CHC model of intelligence, outlined in McGrew (2009), should not be considered the final conceptualisation of the dimensional nature of intelligence. Instead, the CHC model is a framework that can be expanded with the possible inclusion of additional dimensions (see Schneider & McGrew, 2018 and Wilhelm & Kyllonen, 2021), including face processing ability (see Meyer et al., 2021).

### Positive manifold between face processing abilities

There is also some evidence for positive inter-associations between face processing abilities, although most studies have estimated the association between only two face processing abilities. For example, Palermo et al. (2013) reported a moderate, significant correlation (*r* = .40; *N* = 80) between face memory, measured by the CFMT, and expression recognition, measured by an emotion matching task. Similarly, Gignac et al. (2024) investigated the inter-association between face perception and expression recognition ability with the CFPT and the Reading the Mind in the Eyes Test (Baron-Cohen et al., 2001a), respectively, and found that face perception and expression recognition correlated at *r* = .42 (*N* = 10,840).

To date, little research has estimated the association between three or more of these key face processing abilities (detection, identity perception and recognition, and expression recognition). For example, Verhallen et al. (2017) and McCaffery et al. (2018) investigated the inter-association between three measures of key face processing ability: the Mooney Face Test, a measure of face detection; the GFMT, a measure of face perception; the CFMT, a measure of face memory. These studies reported positive correlations, ranging from *r* = .10 to *r* = .48, providing evidence for a general face processing ability factor (*f*).

We note that the *f* factor appears to account for approximately 25% of the variance in the test scores, a value less than that reported for general intelligence (i.e., 50%), at least when measured with non-range restricted samples (Deary, 2001). Like typical Stratum II cognitive abilities, the magnitude of the positive correlations between face processing abilities suggests the presence of a positive manifold (and a common factor); however, each face processing ability appears to be somewhat distinct. The research in the area to date is limited. First, no study has yet measured all four face processing abilities (detection, perception, memory, and expression recognition) simultaneously. Furthermore, previous studies were based on either small or relatively range restricted samples (i.e., university students). Finally, no study has yet employed latent variable modelling to estimate the true score correlations between three or more of the key face dimensions (detection, identity perception and memory, and expression recognition); thus, the reported correlations to date are likely underestimates. This investigation aimed to estimate, in a relatively large community sample, the true score inter-associations between four face processing abilities (via latent variable modelling), including investigating the possibility of the presence of *f*.

### Brief review of the current empirical findings into face processing and intelligence

A positive association between face processing abilities and cognitive abilities more generally may be theoretically expected. Meyer et al. (2021) conducted a review to demonstrate how face-specific abilities can be integrated into the nomological net of socio-cognitive abilities and intelligence. They suggested that face perception and face memory can be integrated into the CHC model of intelligence as a *visuospatial ability* (*social*) and *short-term memory* (*social*) stratum II abilities. However, current evidence linking face processing abilities with intelligence is limited and inconsistent, hindered by narrow sample ranges (e.g., university students) and inadequate intelligence measures (Walker et al., 2023a). Gignac and Bates (2017) provided intelligence measurement guidelines, highlighting test duration, the diversity of subtests, and the range of intelligence dimensions. Poor measures often involve brief testing (less than 10 min) and limited scope, focusing on a single dimension.

To date, a limited number of studies have explored the link between face detection ability and cognitive skills. Walker et al. (2023b) observed a moderate positive correlation (*r* = .44, *N* = 263) between the Mooney Face Test (short-form) and general flexibility of closure. Vigen et al. (1982) reported a weaker positive correlation (*r* = .25) with the WAIS-R Full-Scale IQ, while McCaffery et al. (2018) found no significant correlation (*r* = .06) using a card sorting task, which may be less indicative of intelligence. Vigen et al. (1982) arguably provided the most reliable effect size estimate between face detection and general intelligence, though their findings might be underrepresented due to the absence of latent variable modelling.

With respect to the association between intelligence and face perception, Walker et al. (2023a) reported a psychometric meta-analytic correlation of *r* = .42, based on 11 studies. Connolly et al. (2019) reported a significant association (β = .48) using latent variable modelling, although their measure of intelligence (i.e., the Cattell Cultural Fair Intelligence Test; Cattell, 1973) would be considered a “poor” to “fair,” according to the Gignac and Bates (2017). Similarly, Wilhelm et al. (2010) reported a significant latent positive association (β = .56) between custom measures of face perception and a variety of intelligence measures (fluid reasoning, memory updating, rotation span, and immediate and delayed memory), based on a community sample (*N* = 209). Although Wilhelm et al. (2010) and others employed generally sound methodologies (good samples and latent variable modelling; e.g., Hildebrandt et al., 2015; Olderbak et al., 2015; Wilhelm et al., 2010), they tended to measure general intelligence with a disproportionately large number of memory-based tests. Thus, to provide a more valid effect size estimate, further research is needed with a more balanced cognitive ability test battery.

Compared with other face processing abilities, more studies have investigated the association between face memory and intelligence. Based on 23 samples, Walker et al. (2023a) reported a psychometric meta-analytic correlation of *r* = .26 between intelligence and face memory. Two examples of these studies, Gignac et al. (2016) and Anstey et al. (2002) reported positive, significant, latent variable associations (*r* = .35 and *r* = .49, respectively) between intelligence and face memory. These studies, however, are limited in that they used range restricted samples (either university students or seniors). Furthermore, Anstey et al. (2002) was based on only 90 participants. Thus, a more valid estimate of the true effect size between face memory and general intelligence may be obtained using latent variable modelling, in combination with good quality general intelligence measurement and a community sample.

Finally, Schlegel and colleagues (2019) meta-analysed 17 studies and reported a modest correlation (*r* = .19) between general intelligence and expression recognition. The correlation was consistent across crystallised intelligence (*r* = .18, *k* = 86), fluid reasoning (*r* = .17, *k* = 52), and visuospatial ability (*r* = .12, *k* = 13). As one example, Lewis et al. (2016) reported a positive and significant correlation (*r* = .15) between their measure of intelligence and face expression recognition. However, as reported by Schlegel and colleagues (2019), the age-restricted samples likely limited representativeness, and low reliability in emotion recognition tasks (α = .62) coupled with rarely reported intelligence task reliability (less than 12%) suggests these correlations may be underestimates.

In summary, to the authors’ knowledge, there has been no investigation into the association between intelligence and all four key face processing abilities in one study. We aimed to investigate the association between intelligence and all four key face processing abilities, based on respectable measurement of intelligence, as well as a relatively representative sample. Given that the CHC model of intelligence is under-inclusive and face processing has been identified as a possible candidate ability (see Meyer et al., 2021; Walker et al., 2023a), the observation of a non-negligible positive association between these key face processing abilities and general intelligence may help further support the inclusion of individual differences in face processing within the CHC model of intelligence. That is, Schneider and McGrew (2018) stated that a positive association between a cognitive ability and general intelligence is one of six criteria for a cognitive ability’s inclusion in the CHC model.

### Diagnosis of developmental prosopagnosia

Prosopagnosia can be acquired, generally involving neurological damage, or developmental or congenital in nature, whereby there is no reported or clearly evident brain injury, or low-level visual deficits (Murray et al., 2018). Developmental prosopagnosia has been reported to affect around two to three percent of the adult neurotypical population (Bowles et al., 2009; Kennerknecht et al., 2008). Diagnosis of developmental prosopagnosia is typically based on the traditional cognitive neuropsychological approach for detecting deficits, i.e., scoring 1.96 *SD*s (generally rounded to 2*SD*s) below the mean on measures of face memory, namely the CFMT (Burns et al., 2022). Tests of familiar face recognition and/or face identity perception are also commonly used as part of diagnosis batteries (see Dalrymple & Palermo, 2016 for review). It has been suggested that commonly used diagnostic protocols may be missing a large proportion (as large as 50%–60%) of individuals who are believed to have developmental prosopagnosia (Bate et al., 2019; Burns et al., 2022). Therefore, improving the diagnostic protocol for prosopagnosia is needed. Murray and Bates (2020) recommended that different versions of the CFMT be administered on two separate days (e.g., CFMT original and CFMT-Aus; McKone et al., 2011). Burns et al. (2022) recommended the prosopagnosia index, a self-reported questionnaire, be administered alongside the CFMT. These recommendations, however, have some notable limitations. For example, re-test effects may affect the CFMT, whereby performance may increase with subsequent testing sessions (see Scharfen et al., 2018). Furthermore, some research argue that people do not have accurate insight into their own face recognition abilities, potentially limiting the benefits of adding a self-report measure to the diagnostic protocol for developmental prosopagnosia (Bindemann et al., 2014; but see Palermo et al., 2017 for opposing findings).

As suggested by Walker et al. (2023a), establishing face processing as a cognitive ability may facilitate the conceptualisation of impairments in face processing abilities (e.g., developmental prosopagnosia) as learning disabilities. A learning disability can be defined as a substantial difference between a specific ability and general intellectual functioning (Wilmshurst, 2012). For example, dyslexia is operationally defined as a substantially lower performance (e.g., 2 *SD*s) in reading ability (a specific ability) and general intellectual functioning (Vellutino et al., 2004). Thus, if a moderate, positive association between face memory and general intelligence is observed, it would suggest the possibility of operationalising developmental prosopagnosia as a learning disability and, thus, provide a potentially more effective diagnostic protocol for this condition.

### Trait-autism, face processing abilities, and intelligence

Trait-autism, also known as the Broader Autism Phenotype, is the distribution of milder forms of traits associated with autism^3^ observed in the neurotypical population (Baron-Cohen et al., 2001b). Three dimensions have been identified by large factor analyses (Baron-Cohen et al., 2001b; Russell-Smith et al., 2011): (1) communication deficits, (2) social abnormalities, and (3) attention to detail and patterns. The communication dimension relates to mindreading and nonverbal communication, such as difficulties in understanding others’ intentions and reduced recognition of nonverbal cues. The social dimension is characterised by a lack of interest in reciprocal social interaction, tendencies to focus more on their special interests during conversation, and difficulties in adaptive behaviours relevant to various social situations. Finally, attention to details and patterns is a non-social dimension characterised by an individual’s detail-orientated processing style, such as noticing patterns, small sounds or details that others may not, as well as a fascination with numbers or collecting information about categories of things. Often, the details and patterns subdimension does not correlate with the other two dimensions, suggesting that a total Autism Quotient (AQ) score of trait-autism should not be calculated or interpreted (Dworzynski et al., 2009; English et al., 2021).

There are substantial individual differences in trait-autism (Baron-Cohen et al., 2001b; Constantino & Todd, 2003) and these traits are highly stable over time (Robinson et al., 2011). As we describe below, there is both theoretical and empirical evidence to suggest that face processing abilities may be associated with trait-autism, whereas intelligence is less likely to be associated with trait-autism.

#### Trait-autism and face processing abilities

The social motivation theory of autism implies that difficulties in face processing experienced by autistic people, specifically face memory and face expression recognition, are a result of reduced interest in viewing faces and, thus, more limited experience with faces (Chevallier et al., 2012; Dawson et al., 2005). Experience with faces does play a critical role in the adequate development of face processing ability (see Pascalis et al., 2011), but it is less clear whether more or less experience affects ability. Furthermore, it is widely accepted that autism, or trait-autism specifically, is a continuum in the general population, with more elevated levels of trait-autism bridging the gap to clinically diagnosed autism (Constantino & Todd, 2003). The social motivation theory of autism is theoretically relevant to trait-autism, specifically the nonverbal communication dimension. That is, as faces are an important source of nonverbal communication, deficits in nonverbal communication may reduce an individual’s motivation to seek out nonverbal cues from the face. A tendency of reduced seeking of nonverbal cues may result in missing important expression/identity cues necessary for expression and identity recognition ability, though other face processing abilities may be impacted negatively as well. Stated simply, diminished face processing abilities may be a consequence of lowered social motivation, which is often related to challenges in nonverbal communication commonly observed in individuals with autism-like traits.

There is some empirical research that may support the theory. For example, Lewis et al. (2016) found that trait-autism correlated negatively with face emotion recognition ability (Study 2); however, when controlling for general intelligence, the association was no longer significant. As a limitation, Lewis et al. (2016) calculated a general trait-autism sum score (*α* = .51), contrary to psychometric recommendations (see English et al., 2021).

Considering both theory and empirical research, it is likely that the more social subdomains of trait-autism, such as nonverbal communication or social skills (specific subdomains measured by the AQ), are the driving force behind previously reported negative associations between face processing ability and trait-autism (e.g., English et al., 2024; Lewis et al., 2016; Valla et al., 2013; Verhallen et al., 2014). In support of this notion, English et al. (2024) found across two experiments that individuals with higher autistic-like communication difficulties, measured with a five-dimension trait-autism questionnaire, demonstrated reduced configural processing of face stimuli, as measured by the Mooney Face test. Similarly, Gignac et al. (2024) measured each subdomain of trait-autism separately, utilising the three-dimension AQ. They found that poorer face perception ability uniquely predicted difficulties in autistic-like nonverbal communication (β = −.21), controlling for verbal intelligence and emotion recognition ability, in a sample of 318 community-based adults. Thus, it may be that only the nonverbal communication dimension of trait-autism associates with face detection and face perception. With respect to face memory ability, Davis et al. (2017) found that higher levels in the social dimensions (social interaction and communication) of the AQ (i.e., greater difficulties) were associated significantly with poorer face memory; however, the effect was significant for females only. A limitation of Davis et al. (2017) is that a small, range restricted sample was used (i.e., university students; *N* = 88). In addition, autistic-like nonverbal communication was not analysed separately from the social interaction dimension. Finally, difficulties in autistic-like nonverbal communication dimension have been found to be associated with poorer performance in labelling of facial expressions (*r* = −.29) and matching of facial expressions (*r* = −.24; Bothe et al., 2019). Although these studies highlight the inter-association between face processing abilities and autistic-like nonverbal communication, there has been mixed findings.

Palermo et al. (2018) failed to find a significant association between trait-autism (AQ total score) and two measures of expression recognition: emotion matching task (*r* = .04) and emotion labelling task (*r* = −.12) in 87 predominantly university students. In addition, Alharbi et al. (2020) failed to identify a significant association with the same measures in a larger sample of 160 university students. The findings of these studies may have been limited by sample sizes and/or sample representativeness, as well as the use of total AQ scores, rather than separate subtests, considering the dimensions of the AQ do not all inter-correlate (English et al., 2021).

To date, it is unclear whether there is a significant association between face processing abilities and the nonverbal communication dimensions of trait-autism. Therefore, latent variable modelling with multiple measures of face processing ability, a reasonably large, more representative sample, and consideration for the multi-dimensionality of trait-autism, is needed to help evaluate the nature and size of the hypothesised associations.

#### Trait-autism and intelligence

Theoretical and empirical research on the link between intelligence and trait-autism has been limited and inconsistent. Early studies, such as those by August et al. (1981) and Minton et al. (1982), indicated below-average intellectual functioning in siblings of autistic children, presumed to have higher levels of trait-autism. However, this finding was not replicated in later research, with Dawson et al. (2007), Folstein et al. (1999), and Lainhart et al. (2002) reporting average intelligence in autistic children’s family members. Notably, only Dawson et al. (2007) and Lainhart et al. (2002) used a trait-autism measure for parents, focusing on the Broader Autism Phenotype. These studies did not explore the trait-autism and intelligence link in the general population but rather compared autistic children’s family members with unassessed controls. Kunihira et al. (2006) did extend this research to a more valid context (i.e., not parents), but failed to find a significant correlation between trait-autism and cognitive ability in Japanese university students, using the Wisconsin Card Sorting Task (Heaton et al., 1993) and the Embedded Figures Test (Witkin et al., 1971). However, their study’s small, homogeneous sample and questionable intelligence measures (Gignac & Bates, 2017) limit its generalisability. Although Lewis et al. (2016) administered an intelligence test and a measure of trait-autism, they did not report the correlation.

Overall, the results suggest that there may be no association between trait-autism and intelligence. More research clearly is needed with directly assessed participants and relatively representative samples. From a practical perspective, if face processing abilities are associated with trait-autism and also intelligence, yet intelligence is not associated with trait-autism, then it is possible that individual differences in intelligence will suppress the effect between face processing abilities and trait-autism (see Paulhus et al., 2004). Thus, controlling for intelligence, the association between face processing ability and trait-autism would be hypothesised to increase. By contrast, if both face processing abilities and intelligence are inter-correlated positively, and both are correlated negatively with trait-autism, it would suggest the possibility of face processing ability as a mediator.

### Summary and purpose

We aimed to investigate whether the nonverbal communication of trait-autism was associated with expression recognition, as well as the other face processing abilities, using a relatively more representative sample and respectable measurement of general intelligence and trait-autism. To the authors’ knowledge, there is no published research on the association between all four face processing abilities and autistic-like nonverbal communication, simultaneously. We aimed to investigate whether the association between autistic-like nonverbal communication and face processing abilities will increase when controlling for general intelligence, considering that general intelligence is unlikely to relate to this dimension of trait-autism, but will positively relate to face processing abilities (i.e., may yield a classical suppressor effect; Paulhus et al., 2004).

This study was preregistered (link below) with the following three hypotheses: (1) it was hypothesised that a positive manifold will be observed based on identity-based (face detection, face perception, and face memory) and emotion-based face processing abilities, suggesting the presence of a general face factor (*f*). (2) It was hypothesised that intelligence will be associated positively with face processing abilities. The observation of moderate, positive associations between general intelligence and face processing abilities may help to establish face processing within the current models of intelligence (e.g., the CHC model of intelligence). and (3) It was hypothesised that the nonverbal communication dimension of trait-autism would be negatively associated with one or more of the face processing abilities. The observation of unique effects between face processing and autistic-like nonverbal communication (controlling for intelligence) would be consistent with the social motivation theory of autism and may facilitate the postulation that difficulties in nonverbal communication could cause deficits in face processing abilities. By contrast, the absence of any significant effects would suggest no possibility of a causal effect.

## Method

### Sample

A total of 254 participants were recruited via the TestableMinds platform. To help minimise confounding variables, participation was restricted by the following inclusion criteria: (1) age between 18 and 49 years, as cognitive abilities are relatively stable within this range; (2) residence in one of the following countries: Australia, the United States, the United Kingdom, New Zealand, Canada, or Ireland, as these countries share linguistic and cultural similarities; and (3) English as their first language.

Each participant was paid $6 USD for participation. Only participants who completed the experiment were included in the analyses.^4^ Half of the data for one participant were missing due to an unknown data encoding error; therefore, this participant’s data were removed from all analyses. The data from 253 participants (120 females, 129 male, 4 other; age *M* = 32.71 years, *SD* = 8.54, range = 18 to 49) were analysed. The primary reported ethnicity was White (79%), and the primary reported nationality was the United Kingdom and Ireland (64%), followed by American and Canadian (21%), Australian (4%), and other nationalities (11%). The participant’s education levels were Master’s/Doctorate (21%), Bachelor’s degree (39%), College/Technical school (19%), high school (19%), and less than high school (2%). A power analysis conducted in Jamovi (The Jamovi Project, 2023) determined that a sample size of 253 participants was associated with power of 89% to detect typical effect size of *r* = |.20| (Gignac & Szodorai, 2016) as significant (*p* < .05).

### Materials

#### The Mooney Face Task, short version (Walker et al., 2023b)

Individual differences in face detection ability were measured using a recently developed psychometrically robust Mooney Face test that takes less than 4 min to administer. The images in the short-form were derived from the Verhallen and Mollon (2016), which have improved image quality compared with the original Mooney Task (Mooney, 1957). For all 48 trials, participants are presented with three degraded black and white images. One image contains a face, whereas the other two are distractors. Participants are given 5 s to select which image has the face using the 1, 2, or 3 key on their keyboard. Higher scores on this measure indicate better face detection ability. Validity evidence in support of the Mooney Face Task includes the observation that it loads positively on a general face processing ability factor (McCaffery et al., 2018; Verhallen et al., 2017), and it activates brain areas specifically involved in face processing when a Mooney Face is consciously perceived (Andrews & Schluppeck, 2004; Kanwisher et al., 1998; Rossion et al., 2011). Moreover, Mooney faces prompt the N170 event-related potential component, which is uniquely responsive to faces (George et al., 2005; Latinus & Taylor, 2005; Towler et al., 2016). In addition, Mooney face images induce the face inversion effect, further confirming engagement of the face perception mechanism (Towler et al., 2016). Theoretical raw score minimum and maximum: 1 to 48. Higher scores on this task indicate better face detection ability. The test scores demonstrated good internal consistency reliability in this sample (ω = .88).

#### Cambridge Face Perception Test – 40s version (CFPT-40s; Duchaine et al., 2007)

Individual differences in face perception were measured using the CFPT-40s. This version of the CFPT is designed to be administered in a shorter duration (40 s per trial) than the full version (60 s). It also excludes the inverted-faces subscale of the original version. There are eight test trials and one practice trial. Participants are presented with a target image of a face, in addition to six, somewhat different, face images in a grid. The participants are required to sort the face images in the grid from most similar to least similar to the target face. This measure is scored by calculating the sum of deviations from the face images correct position. Theoretical raw score minimum and maximum: 1 to 144. Higher scores on this measure usually indicate poorer face perception performance; however, for the purposes of this investigation, the scores were reflected so that higher scores indicated better performance. The test scores demonstrated acceptable internal consistency reliability in this sample (ω = .77).

#### Cambridge Face Memory Test (CFMT; Duchaine et al. 2006)

Individual differences in face memory were measured using the CFMT. First, participants must learn six novel faces from three different perspectives (front, left side, and right side). The next phase of the task is the test phase where participants must complete 30 trials. Three images of faces are presented, but only one image contains a learnt face from the previous phase. Participants are required to press 1, 2, or 3 on their keyboard to make their selection. The original version has one practice trial, 18 learn trials, 30 no-noise trials, and 24 noise trials. Previous research has suggested that removing the noise section of the CFMT produces a quicker measure and has essentially equal validity (Corrow et al., 2018; Murray & Bate, 2020). Therefore, the noise section was excluded from the task. The CFMT is scored by summing the number of correct trials on the 18 learn trials and the 30 no-noise trials (theoretical raw score minimum and maximum: 1–48); thus, higher scores indicate better face memory ability. The test scores demonstrated good internal consistency reliability for this sample (ω = .83).

#### Cambridge Car Memory Test (CCMT; Dennett et al., 2012)

This measure is designed to be a non-face comparison task for the CFMT. Its primary use is to control for general object recognition ability, as it is matched in format, structure, and general cognitive requirements of the CFMT. The only difference in the two tasks is that the CCMT asks participants to recognise cars instead of faces. The noise section was also removed in this task to match the CFMT for this investigation. Scoring is the same as the CFMT, with higher scores indicating better general object recognition ability. The test scores demonstrated acceptable internal consistency reliability (ω = .72)

#### Emotion Labelling Task – 48 items (as listed in Palermo et al., 2013, supplementary materials)

Individual differences in expression recognition were measured by an emotion labelling task. This task uses images of posed facial expressions. Participants view the images and are asked to identify the emotion displayed by clicking on one of the six options (anger, happiness, disgust, sadness, fear, or surprise). We used a 48-item version. Theoretical raw score minimum and maximum: 1 to 48. Higher scores indicate better expression recognition performance. The test scores demonstrated acceptable internal consistency reliability (ω = .76).

#### Concrete and Abstract Words Synonym Test (Warrington et al., 1998)

Crystallised intelligence was measured using a vocabulary task. This test has two parts: 25 concrete target words (English words that refer to tangible or physical entities, e.g., shed, labyrinth) and 25 abstract target words (English words that represent intangible concepts or ideas, e.g., pallid, garrulous). For each test question, a participant is presented with a target word and two alternatives from which to choose (5 s to respond). One of the alternatives is a synonym of high frequency and the other words is a distractor of similar frequency. Theoretical raw score minimum and maximum: 1 to 50. Higher scores on this test indicate better crystallised intelligence. The test scores demonstrated good internal consistency reliability (ω = .80).

#### Paper Folding Test (Ekstrom et al., 1976)

Visuospatial ability was measured with the paper folding test. For each test item (20 in total), participants are presented with figures on the left, representing the steps of folding a piece of paper, and the end step consistent with a hole punched into the thickness of the folds. The right side shows the five response alternatives for what the paper might look like once unfolded, only one alternative is correct. Theoretical raw score minimum and maximum: 1 to 20. Higher scores on this task are indicative of higher visuospatial ability. The test scores demonstrated good internal consistency reliability (ω = .83).

#### Visual Working Memory Test

Memory span was measured with a newly developed visual working memory test. Each test trial includes three stages. First, participants are asked to memorise one or more figures (non-descriptive shapes) for 7 s. The number of figures in this first stage increases as the trials progress, starting with one figure and increasing to six. The second stage is a distractor stage, where participants must complete a simple arithmetic problem. The final stage of the trial is a test stage, where participants are shown five figures and asked to select which figure was previously seen in the memorisation stage. As per digit span from the Wechsler scales (Wechsler, 1945), there are two trials per test item. Therefore, participants are exposed to a total of 12 memorisation trials (i.e., two trials to remember and recall one figure, two trials to remember and recall two figures, etc.). No image was used more than once in the task to ensure each trial is unique. This measure is scored by number of correct recalls (theoretical raw score minimum and maximum: 1–12), and a higher score indicates better memory span ability. The test scores demonstrated relatively low internal consistency reliability (ω = .54).

#### Baddeley’s Semantic Verification Test – Forms A and B (Baddeley, 1968)

Individual differences in speeded reasoning were measured with the Baddeley’s Semantic Verification Test (Forms A and B). According to the CHC model, speeded reasoning is considered a narrow, specific ability that falls under the broader domains of fluid intelligence and processing speed (Schneider & McGrew, 2018). In this task, participants are given a short statement in reference to letters A and B. The participant needs to decide if the statement is true or false as quickly as possible. An example of a true statement is “A follows B: BA.” For each form, participants are given a maximum of 75 s (theoretical raw score minimum and maximum: 1–64). Higher scores on this test indicate better speeded reasoning ability. The test scores demonstrated excellent internal consistency reliability (ω = .95).

#### Autism Spectrum Quotient (AQ; Baron-Cohen et al., 2001b)

Trait-autism was measured using the 28-item version of the Autism Spectrum Quotient (Russell-Smith et al., 2011). This questionnaire measures three dimensions of trait-autism: deficits in communication/mindreading, poor social skills, and attention to detail. This measure uses a 5-point Likert-type scale for each question, ranging from “*strongly disagree*” (1) to “*strongly agree*” (5). For our investigation, each dimension showed acceptable to good reliability: communication (ω = .82), social (ω = .87), and attention to detail (ω = .74).

### Procedure

Participants were able to access this study online using their active TestableMinds account and a compatible device, such as a computer desktop or laptop. An informed consent form needed to be read and accepted before proceeding to a calibration screen, which ensured all images and questionnaires were presented in the correct size and format. The first instructions were for participants to minimise any distractions, such as silencing their phone, and position themselves arm’s length from the screen without slouching. They were reminded to also wear any required corrective eye wear. Participants completed demographic questions, followed by the AQ, Mooney Face Test (short-form), CFPT-40s, CFMT, CCMT, Emotion Labelling Task, Visual Working Memory Test, Baddeley’s Semantic Verification Test Form A & B, Paper Folding Test, and ending with the Concrete and Abstract Words Synonym Test. Participants were also asked an attention question after the first task, the Mooney Face Test (short-form), during the middle of the study after the Emotion Labelling Task, and at the end of the study after the Concrete and Abstract Words Synonym Test: “To show us that you were paying attention, can you explain in one sentence what we asked you to do in this part of the study?.” The study ended with a final question about whether there was any reason not to use their data in this study; for example, technical issues, lack of motivation, or not understanding the instructions. The average completion time was 53.96 minutes. This study’s procedure was approved by the University of Western Australia’s Human Research Ethics Committee (2019/RA/4/1/6704). This study was submitted for preregistration and is available online at https://osf.io/vh42f.

### Data analysis

All analyses were conducted in Jamovi, SPSS, and SPSS-Amos (Arbuckle, 2023; The Jamovi Project, 2023). Pearson correlations between all key observed variables were estimated. A latent variable and structural equation modelling strategy was employed to evaluate the association between face processing abilities, intelligence, and autistic-like nonverbal communication, using the statistical software SPSS-Amos. All model solutions were estimated via maximum likelihood, and statistical significance was determined via bootstrapping (bias-corrected accelerated; 2,000 resamples). Several close-fit indices were consulted for evaluation of model and close fit; specifically, root mean square error of approximation (RMSEA), standardised root mean square residual (SRMR), Tucker–Lewis index (TLI), and comparative fit index (CFI). For the purpose of identification/scaling for all models, each exogenous latent variables’ variance was fixed to 1; for endogenous latent variables, a factor loading was fixed to 1. Across all models, an item parcelling strategy was employed. That is, three composite scores were created for each test and used to define the latent variables (see the online supplementary materials for further details).

With respect to latent variable modelling, first, across face processing abilities and intelligence, we tested a series of higher-order, single-factor, and correlated factor models (the latter two are available in supplementary materials). It was expected that the higher-order model for both face processing and intelligence would fit better than the single-factor and possibly the correlated factor models.

Based on the measurement model results, we proceeded to estimate the latent variable effects between face processing abilities, intelligence, and trait-autism (nonverbal communication), consistent with the preregistered models. Specifically, intelligence, general object recognition ability (CCMT), and autistic-like nonverbal communication difficulties were specified to predict face processing abilities. A similar model controlling for age, which was preregistered, was also tested, and reported in the online supplementary materials.

Five additional models were tested. First, a correlated model for face memory and general intelligence was tested to provide support for the potential diagnosis of developmental prosopagnosia as a learning disability. Second, a higher-order model for general intelligence with the inclusion of face processing abilities was analysed. Third, face detection, face perception, and face memory were specified to predict expression recognition. Fourth, a developmental cascade model was tested for the four face processing abilities, specifically face detection → face perception → face memory → expression recognition. Fifth and finally, a higher-order model with face processing abilities predicting general intelligence (reported in the online supplementary materials).

## Results

All data and scripts are available at https://osf.io/yjkf5/.

### Data screening

The total scores for the data were sufficiently normally distributed (skew < 2.0| and kurtosis < |9.0|) for the purpose of parametric analysis (Bishara & Hittner, 2012). No univariate outliers were identified based on the interquartile range outlier labelling rule with a 3.0 multiplier (Hoaglin & Iglewicz, 1987). Five multivariate outliers were detected for the CFPT total scores, based on an examination of Cook’s distance and dfFits values. Consequently, we removed these CFPT scores and estimated them via a missing value analysis (expectation maximisation).^5^

### Descriptive statistics and correlations

Descriptive statistics for all total scores are reported in Table 1. The AQ Communication subscale had a mean score of 2.30 (*SD* = 1.04), implying a mean response between “*Somewhat disagree*” and “*Neither agree nor disagree*.” By comparison, the AQ Social subscale (*M* = 3.01; *SD* = 1.20) and the AQ-Attention to detail subscale (*M* = 2.95; *SD* = 1.17) means were approximately 3.0, implying a mean response of “*Neither agree nor disagree*.” For the face processing tasks, performance varied from respondents correctly sorting, on average, 68.8% (*SD* = 12.7%) of the images for the CFPT to respondents correctly completing, on average, 83.89% (*SD* = 11.54%) of the trials for the CFMT. Likewise, performance varied on the cognitive ability tasks, with respondents correctly completing on average 57.5% (*SD* = 16.3%) of the items for the visual working memory capacity (WMC) test to respondents correctly completing on average 73.2% (*SD* = 11.9%) of the items for the Synonym Test. Finally, there was little evidence of ceiling effects across all tasks, with typically substantially fewer than 5% of participants achieving 100% on the task: Mooney = 2.37%; CFPT = 0%; CFMT = 5.53%; Emotion Labelling = 0%; CCMT = 0.40%; Visual WMC = 0.79%.

**Table 1.** Descriptive statistics for observed total scores and age.

	*M*	*SD*	CV	Min.	Max.	Skew	Kurtosis
Age	32.71	8.54	.26	18.00	49.00	.12	−1.02
AQ Social	39.13	10.31	.26	15.00	64.00	.01	−.49
AQ Comm	18.40	5.52	.30	8.00	37.00	.40	.14
AQ ATD	20.65	5.13	.25	7.00	32.00	−.27	−.45
Mooney Test (%)	70.31	16.673	.24	31.25	100.00	−.03	−.96
CFPT (%)	68.75	12.68	.18	19.44	91.67	−1.17	2.11
CFMT (%)	83.89	11.54	.14	35.42	100.00	−.61	.15
CCMT (%)	66.58	12.49	.19	33.33	100.00	.07	−.37
Emotion Labelling (%)	74.79	10.61	.14	33.33	93.75	−.74	.66
Visual WMC (%)	70.65	14.60	.21	25.00	100.00	−.62	.13
Baddeley’s Reasoning Test (%)	57.51	16.29	.28	18.75	98.44	.04	−.61
Paper Folding Test (%)	54.98	21.51	.39	10.00	95.00	−.19	−.89
Synonym Test (%)	73.16	11.91	.16	40.00	96.00	−.22	−.45

*Note. N* = 253, except for age (one missing value); CV = coefficient of variation; AQ = Autism Quotient; Comm = communication; ATD = attention to detail; CFPT = Cambridge Face Perception Task; CFMT = Cambridge Face Memory Task; CCMT = Cambridge Car Memory Task; WMC = working memory capacity.

As can be seen in Table 2, the observed score inter-correlations between the face processing abilities ranged from .18 to .44 and were statistically significant. The positive inter-correlations between the face processing abilities indicated that better performance on one face processing ability was associated with better performance on the other face processing abilities, i.e., a general face processing factor. Similarly, all correlations between the cognitive abilities were positive and statistically significant, ranging from .21 to .43, implying the presence of a general factor of intelligence.

**Table 2.** Pearson inter-correlations between observed variables and internal consistency reliabilities.

	1.	2.	3.	4.	5.	6.	7.	8.	9.	10.	11.	12.	13.
1. Age	—												
2. AQ Social	.07	(.87)											
3. AQ Comm	−.02	**.52**	(.82)										
4. AQ ATD	−.03	.10	**.16**	(.74)									
5. Mooney	.10	−.02	−.09	−.00	(.88)								
6. CFPT	−.04	−.11	−**.34**	−.08	**.27**	(.77)							
7. CFMT	.09	−.11	−**.31**	−.05	**.33**	**.48**	(.83)						
8. CCMT	**.17**	−.04	−.08	.11	**.20**	**.18**	**.38**	(.72)					
9. Emotion Labelling	.04	−**17**	−**.30**	−.03	**.26**	**.37**	**.44**	**.15**	(.76)				
10. VWMT	.00	.06	−.06	−.08	.08	**.26**	.12	**.23**	**.24**	(.54)			
11. BSVT	−**.15**	.03	−.10	−04	−.01	**.20**	.11	.12	**.25**	**.22**	(.95)		
12. Paper Folding	−.12	.11	−.06	−.06	.09	**.30**	**.21**	**.26**	**.21**	**.38**	**.43**	(.83)	
13. Synonym Test	**.32**	.09	−.03	−.11	**.14**	**.18**	**.19**	**.29**	**.21**	**.26**	**.40**	.**37**	(.80)

*Note. N* = 253; bold = *p* < .05; McDonald’s omega is displayed on the main diagonal in parentheses; the McDonald’s omega for the CFPT was calculated with the five outlier cases removed; AQ = Autism Quotient; Comm = Communication; ATD = attention to detail; CFPT = Cambridge Face Perception Task; CFMT = Cambridge Face Memory Task; CCMT = Cambridge Car Memory Task; VWMT = Visual Working Memory Task; BSVT = Baddeley’s Semantic Verification Test.

Most (70%) of the correlations between the cognitive ability tests and the face processing tests were significant and positive (see Table 2). In particular, we note that the Synonym Test was significantly, positively correlated with all the face processing tasks, implying that higher levels of crystallised intelligence were associated with better performance on all of the face processing abilities. In addition, the Paper Folding test correlated positively and significantly with all of the face processing abilities, except for the Mooney test. These results suggest that general intelligence and general face processing ability are dimensions that are inter-correlated positively, as hypothesised.

Finally, the AQ subscales tended to correlate negatively with the face processing abilities, but consistently in a statistically significant way only for the AQ Communication subscale: CFPT *r* = −.34, *p* < .001; CFMT *r* = −.32, *p* < .001; and Emotion Labelling *r* = −.30, *p* < .001. Such results imply that lower levels of communication difficulties were associated with higher face perception, face memory, and expression recognition performance. By contrast, there were no significant correlations between any of the AQ subscale scores and the general cognitive ability tasks (see Table 2).

### Latent variable measurement models

**Figure 1.**
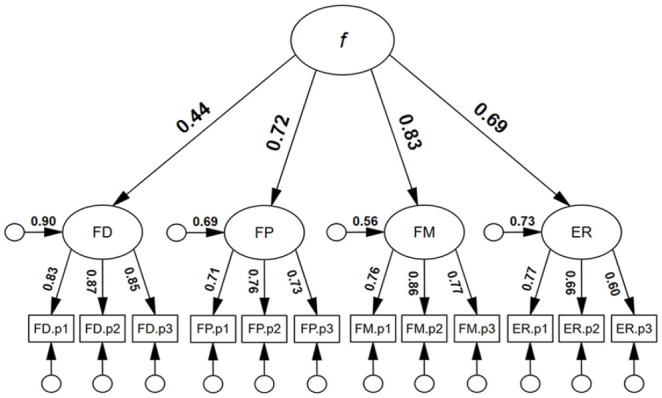
Higher-order four-factor model for face processing. *Note. N* = 253; f = general face factor; FD = Face Detection; FP = Face Perception; FM = Face Memory; ER = Expression Recognition; all coefficients were statistically significant, *p* **<** .05.

First, we tested a higher-order face processing model. As can be seen in Figure 1, all first-order loadings were positive and statistically significant. In addition, all first-order factor residual variances were statistically significant: FD: .90, *p* = .001; FP: .70, *p* = .001; FM: .56, *p* = .002; ER: .73, *p* = .001; suggesting that each dimension of face processing was, at least to some degree, unique. All second-order loadings were positive and statistically significant, ranging from λ = .44 to .83, *p* < .001. The model was associated with excellent model close-fit, χ^2^(50) = 56.55, *p* = .613, RMSEA < .001, SRMR = . 032. TLI = 1.004, CFI = 1.000. By comparison, a single-factor face processing model was not well-fitting, χ^2^(54) = 570.64, *p* < .001, RMSEA = .195, SRMR = .13, TLI = .485, CFI = .579. Finally, a correlated factor face processing model had good model-fit, similar to the higher-order model, χ^2^(48) = 46.37, *p* = .540, RMSEA < .001, SRMR = .032, TLI = 1.002, CFI = 1.000 (see supplementary material, Figures S1 and S2 for full results). On the basis of parsimony, we preferred the higher-order model.

Next, a higher-order model of general intelligence (*g*) was tested and found to have good model close-fit: χ^2^(50) = 68.70, *p* = .041, RMSEA = .039, SRMR = .045, TLI = .983, CFI = .987 (see supplementary material, Figure S3 for more details). All of the first-order factor loadings were statistically significant, and all of the second-order loadings were also statistically significant, ranging from λ = .61 to .79, *p* < .001. Two other models for *g* were tested (see supplementary material, Figures S4 and S5 for full results). Again, on the basis of parsimony, we preferred the higher-order model.

Prior to reporting the structural equation modelling results, we note that latent variable *g* correlated significantly and positively with AQ Social, *r* = .15, *p* = .030 and negatively with AQ Attention to Detail, *r* = −.18, *p* = .046, but non-significantly with AQ Communication, *r* = −.12, *p* = .174 (see supplementary material, Figure S6). The significant correlation suggests that higher levels of intelligence were associated with higher levels of self-reported socialisation difficulties and lower levels of self-reported attention to detail, though the effects were relatively small (Gignac & Szodorai, 2016).

### Structural equation modelling

A model to estimate the association between AQ Communication and *f*, controlling for the effects of general object recognition (ORA) was preregistered for this investigation (see supplementary material, Figure S7). Importantly to note from this model, AQ Communication was uniquely associated with *f*, β = −.46 (before controlling for intelligence). The negatively directed AQ Communication coefficient implied that higher levels of face processing ability were associated with lower levels of nonverbal communication abilities, as hypothesised.

**Figure 2.**
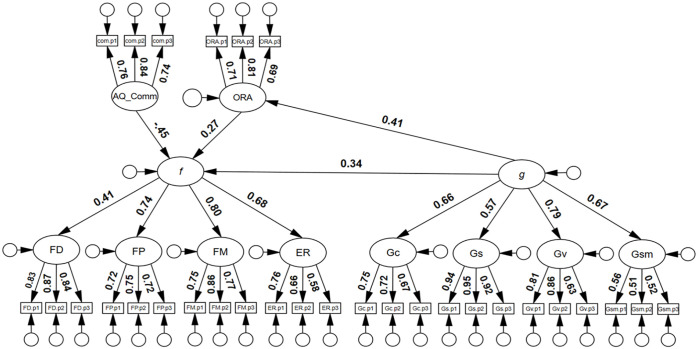
Structural equation model with general cognitive factor predicting the general face factor. *Note. N* = 253; AQ_Comm = AQ Communication; ORA = General Object Recognition; f = general face factor; FD = Face Detection; FP = Face Perception; FM = Face Memory; ER = Expression Recognition; g = general cognitive factor; Gc = crystallised intelligence; Gs = speeded reasoning; Gv = visuospatial ability; Gsm = memory span; all coefficients were statistically significant, *p* **<** .05.

Finally, a model to estimate the inter-association between AQ Communication, *f*, and *g*, controlling for the effects of ORA was tested. As can be seen in Figure 2, *g* was a significant predictor of *f*, β = .34, 95% confidence interval (CI) = [.15, .54], *p* = .001, and the model had good model close-fit: χ^2^(393) = 454.66, *p* = .017, RMSEA = .025, SRMR = .060, TLI = .979, CFI = .981. There was no evidence to suggest that *g* was associated with the AQ communication latent variable. There was also little evidence to suggest that *g* suppressed the association between face processing abilities (*f*) and AQ communication (i.e., the AQ communication beta-weight was very similar, −.46 and −.45, respectively, in both models; see supplementary material, Figure S7 and Figure 2).

An additional model was tested (as preregistered), whereby the effects of age was controlled; however, the results were essentially the same (see supplementary material, Figure S8).

### Additional models

**Figure 3.**
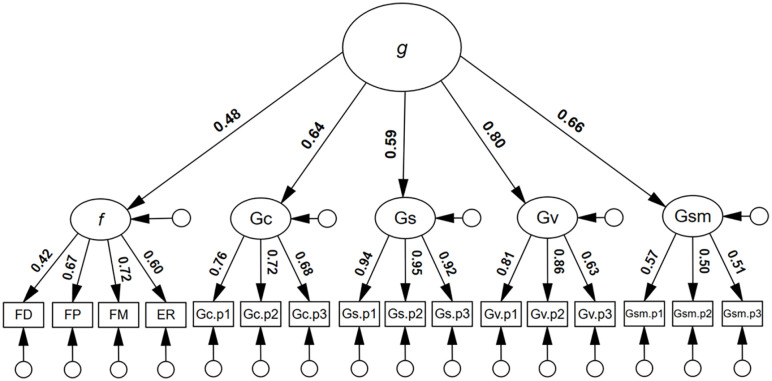
Higher-order model for general intelligence with the inclusion of face processing abilities. *Note. N* = 253; g = general cognitive factor; f = general face factor; FD = face detection; FP = face perception; FM = face memory; ER = expression recognition; Gc = crystallised intelligence; Gs = speeded reasoning; Gv = visuospatial ability; Gsm = memory span; all coefficients were statistically significant, *p* **<** .05.

To estimate the shared variance between *g* and *f*, and the potential position of face processing ability within the CHC model, an additional higher-order model was tested, whereby *f* was modelled under *g* as a first-order factor. As can be seen in Figure 3, *f* yielded a significant first-order factor *g* loading, λ = .49, 95% CI = [.32, .65], *p* = .001, a value that corresponded to 24% shared variance between *g* and *f*, *R*^2^ = .24, 95% CI = [.10, .43]. The model showed excellent model close-fit, χ^2^(99) = 120.541, *p* = .070, RMSEA = .029, SRMR = .052, TLI = .984, CFI = .987.

**Figure 4.**
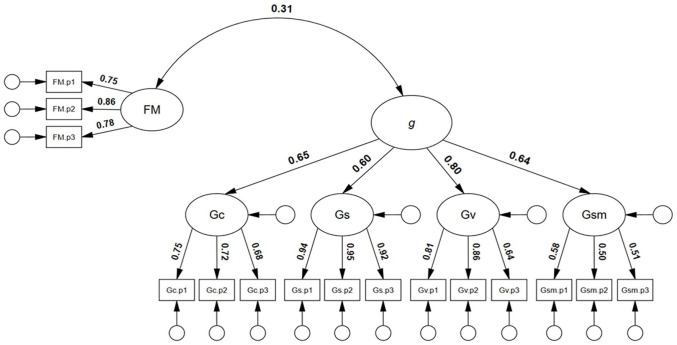
Correlated model of face memory with general intelligence. Note. *N* = 253; FM = face memory; g = general cognitive factor; Gc = crystallised intelligence; Gs = speeded reasoning; Gv = visuospatial ability; Gsm = memory span; coefficients in bold were statistically significant, *p* **<** .05.

Next, and particularly relevant to the potential diagnosis of developmental prosopagnosia, we sought to test whether face memory ability was significantly and positively correlated with general intelligence. As can be seen in Figure 4, face memory was significantly and positively correlated with general intelligence, *r* = .31, 95% CI = [.15, .47], *p* = .001. The model showed good model close-fit, χ^2^(85) = 103.70, *p* = .082, RMSEA = .030, SRMR = .0484, TLI = .987, CFI = .989.

**Figure 5.**
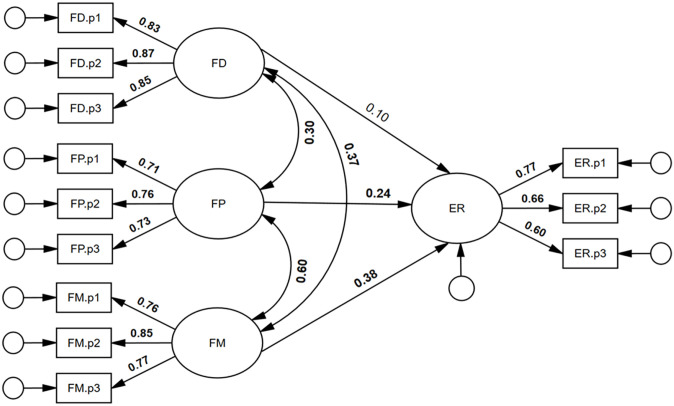
Face detection, face perception, and face memory predicting expression recognition ability. Note. *N* = 253; FD = Face Detection; FP = Face Perception; FM = Face Memory; ER = Expression Recognition; coefficients in bold were statistically significant, *p* **<** .05.

We also sought to test whether face detection, face perception, and face memory were unique predictors of expression recognition ability. As can be seen in Figure 5, face detection was not a significant, unique predictor of expression recognition ability, β = .10, 95% CI = [−.05, .43], *p* = .215. By contrast, both face perception and face memory were significant, unique predictors of expression recognition ability: β = .24, 95% CI = [.04, .43], *p* = .023, and β = .38, 95% CI = [.16, .57], *p* = .002, respectively. It was found that 37% of the variance in expression recognition ability was accounted for by the face processing abilities, *R*^2^ = .37, 95% CI = [.22, .50], *p* = .003. The model had excellent model close-fit, χ^2^(48) = 46.37, *p* = .540, RMSEA < .001, SRMR = .032, TLI = 1.002, CFI = 1.000.

**Figure 6.**
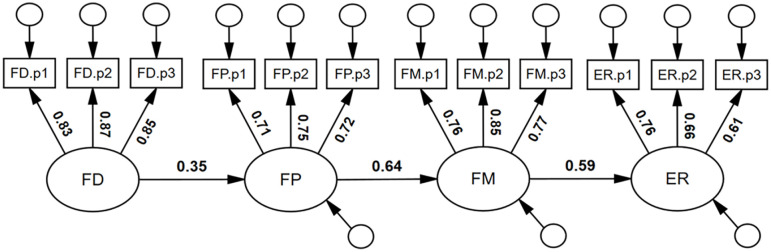
Suggested cascading model of face processing abilities. Note. *N* = 253; FD = Face Detection; FP = Face Perception; FM = Face Memory; ER = Expression Recognition; coefficients in bold were statistically significant, *p* **<** .05.

We note that the pattern of coefficients was such that FD, FP, and FM were incrementally (numerically) larger predictors of ER. Consequently, we tested a cascading model of face processing abilities (see Figure 6), a model that had excellent model-fit, χ^2^(51) = 64.57, *p* = .096, RMSEA = .032, SRMR = .065, TLI = .986, CFI = .989. Face detection was a significant predictor of face perception, β = .35, 95% CI = [.21, .48], *p* = .001; face perception was a significant predictor of face memory, β = .64, 95% CI = [.51, .76], *p* = .001; and face memory was a significant predictor of expression recognition ability; β = .59, 95% CI = [.45, .71], *p* = .001.

Finally, a supplementary model was tested whereby the four face processing abilities were specified as predictors of general intelligence. FP (β = .30) and ER (β = .26) were found to be significant unique predictors (see supplementary material, Figure S9 for further details).

## Discussion

We examined the associations between four primary face processing abilities (face detection, face perception, face memory and expression recognition), general intelligence, and trait-autism (autistic-like nonverbal communication). First, we were able to establish a general face factor (*f*) via the four positively inter-correlated face processing abilities. Second, we found a moderate, positive association between *f* and a general cognitive factor (*g*), providing empirical support for the potential inclusion of face processing abilities within the CHC model of intelligence. Third, based on a latent variable model predicting general face processing, we found that autistic-like nonverbal communication was a significant, negative predictor of face processing ability. Finally, though intelligence failed to associate significantly with autistic-like nonverbal communication, it was also not found to suppress the association between face processing abilities and nonverbal communication ability (i.e., when controlling for intelligence, the association between face processing abilities and nonverbal communication ability did not change).

### Face processing abilities and the general face factor (*f*)

We supported the hypothesis of positive associations between face detection, face perception, face memory, and expression recognition, reflecting a general face factor. The latent variable (true score) inter-associations between the four face processing abilities (Table 2) ranged from .26 (face detection & expression recognition) to .44 (face memory and expression recognition), i.e., relatively small to large effect sizes for individual differences research (Gignac & Szodorai, 2016). Our correlations are comparable to those reported in previous research in this area (e.g., Gignac et al., 2024; Verhallen et al., 2017). The observation of a positive manifold for face processing abilities supports the existence of a general face factor (*f*), as proposed by Verhallen et al. (2017). We extend the research by examining the *f* dimension through latent variable modelling, a method that is not impacted by measurement error (Gimenez et al., 2005).

Based on the latent variable modelling, a higher-order four-factor model had excellent model close-fit, whereas the single-factor model was not well-fitting. There is evidence that, while there is a general face factor, there also appears to be specific face factors (i.e., variance unique to each face processing ability). Our results, therefore, are in line with neuropsychological evidence, which suggest that face processing abilities overlap to some degree. For example, fMRI findings imply that two distinct brain regions, the fusiform face area and the posterior superior temporal sulcus, are sensitive to changes associated with both identity and expression (Fox et al., 2009; Xu & Biederman, 2010). Finally, the higher-order factor model of face processing (Figure 1), with factor loadings that ranged from .44 to .83 (*M* = .67), was consistent with a moderately strong general face factor. The *g* factor in this investigation had an identical mean factor loading of .67 (supplementary material, Figure S3).

Based on the correlated factor model of face processing abilities (supplementary material, Figure S2), the largest true score associations were observed between face memory, face perception, and expression recognition ability (correlations ranging from .50 to .60). The greater shared variance between these three tasks may be due to a greater number of shared underlying higher-order cognitive processes and/or greater similarities in task complexity, as typically interpreted in general factor intelligence research (Jensen, 2006). That is, arguably, the ability to identify, differentiate, and recognise faces and facial emotion expressions may require higher greater cognitive load, in comparison with the detection of a face (e.g., Mooney). However, the degree to which these abilities involve shared or separate functional and neural pathways has been extensively debated (see Calder & Young, 2005 for an overview of the debate). Further research is recommended to gain a better understanding of the common and distinct underlying mechanisms of the face processing abilities.

### The association between face processing and intelligence

Consistent with many other investigations (Carroll, 1993; Gignac et al., 2016; Jensen, 1998; Meyer et al., 2021), we observed a general factor of intelligence (*g*), based on our measures of crystallised intelligence (*Gc*), visuospatial ability (*Gv*), short-term memory span (*Gsm*), and speeded reasoning (*Gs*). Our higher-order model (Figure 5) demonstrated that *g* was a significant positive predictor of face processing abilities (*f*). Thus, the hypothesis that general intelligence would associate with face processing ability was supported. The results of our investigation are consistent with previous research that found a positive association between general intelligence and face detection (e.g., Vigen et al., 1982), face perception (e.g., Connolly et al., 2021; Wilhelm et al., 2010), face memory (e.g., Gignac et al., 2016; Hildebrandt et al., 2011), and expression recognition (e.g., Lewis et al., 2016; Schlegel et al., 2019). Previous research that failed to find significant, positive associations (e.g., Palermo et al., 2013; Shakeshaft & Plomin, 2015; Wilmer et al., 2014) tended to use poorer quality intelligence measures and/or restricted samples, which likely curtailed the variability in cognitive ability test scores.

Correspondingly, based on a meta-regression of the intelligence and face processing literature, Walker et al. (2023a) found that the association between face processing abilities and intelligence was moderated positively by the quality of intelligence measures. Our investigation extends the literature, as we measured intelligence across four dimensions. By comparison, a large number of previous studies in the area included only one or two subtests (e.g., Connolly et al., 2019; McCaffery et al., 2018; Palermo et al., 2013). However, according to Gignac and Bates (2017), the quality of our intelligence measurement quality would be rated as “good,” though not “excellent.” Furthermore, we used a relatively large and more representative sample, compared with previous investigations in the area. Specifically, Walker et al. (2023a) found that only 35% of studies used a general community sample and at least good measures of intelligence, as defined by the Gignac and Bates (2017) criteria for intelligence testing. We encourage researchers to use good to excellent measures of intelligence and opt for general community samples when investigating the association between intelligence and face processing abilities.

As general intelligence and general face processing ability shared 24% of their variance, it implies that face processing ability and intelligence are clearly inter-related but, also, not isomorphic constructs. Similarly, reading comprehension is described as a dimension that shares a respectable amount of variance (approximately 14%) with general intelligence (Peng et al., 2019), but is not considered identical to general intelligence. Instead, researchers conduct a considerable amount of research into the specific construct of reading comprehension, because it has important educational and professional consequences (e.g., Elleman & Oslund, 2019). Therefore, further research on individual differences in face processing ability is encouraged, despite its meaningful association with *g*. Researchers may consider simultaneously administering intelligence tests, to control for the shared variance, and evaluate effects unique to face processing ability, as conducted in this investigate with respect to trait-autism. In some cases, intelligence may mediate effects and in others it may suppress them. Either way, given the non-trivial magnitude of the positive association between *g* and *f*, differential psychology researchers studying face processing abilities may commonly stand to benefit by measuring *g*, to estimate non-confounded effects.

### Implication for the CHC model of intelligence

Walker et al. (2023a) provided a comprehensive theoretical case for the potential integration of face processing within the CHC model of intelligence. Based on the results of their meta-analysis, they recommended further empirical research to examine whether face processing could be conceptualised within the CHC model of intelligence. Our investigation provides further empirical support towards the inclusion of face processing abilities within the CHC model of intelligence. Specifically, we propose that face processing could potentially be a Stratum II (broad) ability within the CHC model of intelligence, with each face processing ability representing a Stratum I (narrow) ability. In support of this, we found evidence that the four face processing abilities measured in this investigation form one coherent construct (factor). This is an important criterion for conceptualising face processing as a potential Stratum II ability.

Our results demonstrate the vast majority of correlations between face processing tasks and cognitive abilities were positive and statistically significant. We note that although there was a non-significant correlation between face detection and our processing speed/speeded reasoning task, processing speed is typically the least *g* loaded dimension (Wechsler, 2008), and we found face detection to be the least *f* loaded task. Thus, these two dimensions may only be associated positively in the population to a relatively smaller degree. Finally, we estimated the association between face processing abilities (*f*) and general intelligence (*g*) at .48 (see Figure 3), a value comparable in size to other candidate abilities for CHC model consideration (e.g., auditory intelligence, λ = .53; emotional intelligence, λ = .69; Bryan & Mayer, 2020), as well as some established CHC model Stratum II abilities (e.g., processing speed, λ = .61; Bryan & Mayer, 2020).

Even though we are proposing face processing for inclusion into the CHC model of intelligence as a Stratum II ability, we acknowledge that face processing abilities may possess greater levels of specificity, in comparison with typical Stratum II abilities included in the CHC model. For example, elements of the brain appear to be uniquely involved with mediating face processing abilities (e.g., Kanwisher & Yovel, 2006), along with possible genetic contributions specific to face identity recognition (Wilmer et al., 2010). For this reason, the face processing ability factor (*f*) may relate to *g* at the lower-end of the spectrum to what is typically observed in the CHC literature for a Stratum II ability.

In addition, although we currently recommend the potential inclusion of a general face factor into the CHC model of intelligence as a Stratum II ability, we acknowledge Meyer et al. (2021) who suggested that face memory and face perception be incorporated into the CHC model as two separate Stratum II abilities. MacCann et al. (2014) proposed that expression recognition be considered as a Stratum I ability under the broader (Stratum II) ability of emotional intelligence. Ultimately, it remains to be determined convincingly whether a general face processing ability should be represented as a respective Stratum II ability, as suggested by this investigation, or whether the facets of face processing ability should be decomposed into distinct Stratum I abilities as indicators of candidate or established Stratum II abilities (i.e., MacCann et al., 2014; Meyer et al., 2021). Additional research is required to answer this question. For example, a potential study could conduct a series of factor analyses to determine how to best represent the correlations between a large battery of cognitive ability and face processing ability task scores. Ideally, it would be essential to measure each established Stratum II ability with three to four subtests, along with subtests designed to measure the commonly investigated four dimensions of face processing (detection, perception, memory, and emotion recognition). Such an investigation would require perhaps two dozen subtests and a large sample size (500+).

### Implications for identifying developmental prosopagnosia

The most commonly utilised diagnostic protocol for developmental prosopagnosia (i.e., 2 *SD*s below the mean on primarily the CFMT) has been criticised for potentially missing a large portion of developmental prosopagnosics; thus, alternative protocols have been suggested (e.g., Burns et al., 2022; Murray & Bate, 2020; Ulrich et al., 2017). Here, we suggest that developmental prosopagnosia be conceptualised as a learning disability, a novel diagnostic perspective for this condition. Currently, the cognitive discrepancy method is the most prominently utilised approach to defining a learning disability. The cognitive discrepancy method operationalises a learning disability as a substantial discrepancy between general cognitive functioning and a specific ability (Fletcher et al., 2013). For example, dyslexia, a reading disability, is defined and diagnosed as substantially lower performance (e.g., 2 *SD*s) in reading comprehension compared with general intellectual functioning (Vellutino et al., 2004). On the basis of their meta-analysis that reported a positive association between intelligence and face memory, Walker et al. (2023a) proposed that developmental prosopagnosia could potentially be diagnosed in a manner similar to other learning disabilities. That is, the cognitive discrepancy method rests upon the observation of an appreciable, positive correlation between general intellectual functioning and the narrower cognitive ability of interest. From this perspective, a face memory deficit, a defining characteristic of developmental prosopagnosia, could be suggested if an individual’s performance on the CFMT is 2 *SD*s or more below that individual’s full-scale intellectual quotient performance (e.g., 2 *SD*s above the mean FSIQ and −1 *SD* below the mean on the CFMT, and −1 *SD* below the mean FSIQ and −3 *SD*s on the CFMT would both be indicative of developmental prosopagnosia). Walker et al. (2023a) recommended further research with better samples and measures to help support this contention. Our results suggest a significant, positive, and non-negligible correlation (.31) between face memory ability and general intelligence, similar in magnitude to the correlation between reading comprehension and general intelligence (*r* = .38; Peng et al., 2019). The findings of this investigation, therefore, help confirm Walker et al.’s (2023a) contention, given the strength of the association between face memory and *g* was comparable to the association between reading comprehension and *g*.

### Autistic-like nonverbal communication, face processing, and intelligence

To the authors’ knowledge, this investigation is the first to examine the association between trait-autism and intelligence with good measures of intelligence and a relatively non-restricted sample. The higher-order model results suggested that autistic-like nonverbal communication ability was not significantly associated with intelligence (*r* = −.12). An additional analysis (supplementary material, Figure S6) found that the social dimension and attention to detail dimensions of trait-autism were significantly correlated with *g* (*r* = .15 and *r* = −.18, respectively). However, these correlations may be considered relatively small in magnitude (Gignac & Szodorai, 2016) and, as such, offer little theoretical or applied relevance. We suggest that these correlations may be due, in part, to the contexts of some of the questions in the AQ. For example, “I would rather go to a library than a party,” an item from the social dimension, arguably measures more of an “intellect” personality dimension, which is known to correlate positively with intelligence (DeYoung, 2015). Overall, based on our results, it would appear that the association between intelligence and trait-autism is likely small to near zero, which is consistent with the findings of other studies with smaller samples and less comprehensive measures (e.g., Dawson et al., 2007; Kunihira et al., 2006).

In contrast to intelligence, face processing abilities were negatively associated with autistic-like nonverbal communication ability. Specifically, autistic-like nonverbal communication was related to *f* relatively strongly at *r* = −.45, controlling for age, general object recognition, and intelligence (Figure 2). Thus, the hypothesis that autistic-like nonverbal communication would relate to one or more of the face processing abilities was supported. Our results are consistent with previous studies that found individuals with more autistic-like nonverbal communication difficulties to have poorer expression recognition (Bothe et al., 2019; Lewis et al., 2016; Poljac et al., 2012) and face memory (Halliday et al., 2014), though our estimated effect size (−.45) appears to be larger than those previously reported (−.20 to −.29). Given our research methods (multiple measures; structural equation modelling), we believe our study provides a robust estimate between multiple face processing abilities and autistic-like communication.

The negative association between autistic-like nonverbal communication difficulties and face processing abilities is consistent with the social motivation theory of autism (Chevallier et al., 2012; Dawson et al., 2005). Specifically, difficulties in autistic-like nonverbal communication could reduce motivation to seek out nonverbal cues from the face and/or reduce engagement in social interaction, subsequently missing important facial expressions and facial identity cues and/or limiting the experience with faces. The limited experience could negatively impact the development of accurate identity and expression perception/recognition. Future research, potentially using eye-tracking equipment, ideally longitudinally spanning a sensitive period of development for face processing (see Pascalis et al., 2020) could further investigate the developmental connection between autistic-like nonverbal communication difficulties and deficits in face processing.

Finally, our findings may be applicable beyond individuals with trait-autism. Autism Spectrum Disorders and trait-autism share more than just symptomatology, and likely have overlapping genetic and biological aetiology (Bralten et al., 2018). Therefore, the findings in this investigation may extend to research into autistic adults. We encourage research to evaluate whether the negative association observed in this investigation between autistic-like nonverbal communication and face processing abilities is present in autistic adults.

### The cascading model of face processing

A supplementary model was tested in investigation to determine whether expression recognition ability was uniquely predicted by each of the other face processing abilities. Our results showed that expression recognition was significantly predicted uniquely by face perception and face memory, but not face detection. Furthermore, face memory yielded a numerically larger beta-weight onto expression recognition than face detection ability, suggesting the possibility of a cascading model of face processing abilities.

A cascading model has been suggested in the area of emotional intelligence (Joseph & Newman, 2010). In this model, emotional intelligence is seen as a process that begins with the most basic psychological processes and moves towards more complex psychological processes. For example, the ability to accurately perceive emotions in oneself and others is foundational and can influence the next level of the cascade, such as understanding and analysing emotions and their effects. As one moves up the cascade, the emotional skills become more complex, culminating in the management of emotions in the self and others (Joseph & Newman, 2010; Shao et al., 2015). A cascading model has also been proposed and supported empirically in the area of intelligence. Specifically Fry and Hale (1996) found that increased processing speed results in improvements in working memory, which, in turn, leads to the improvement of fluid intelligence.

Face processing abilities could be conceptualised in a similar way. That is, theoretically, each face processing ability may be considered to be associated with different cognitive loads and complexities. For example, the relatively simple process of detecting a face in an image could be argued to require substantially fewer cognitive resources to execute successfully, in comparison with recalling a previously presented face at a slightly different angle. During infancy, face detection is likely the first ability to develop before any further face processing ability, such as identity or expression recognition (Pascalis et al., 2011). Thus, at least tentatively, the following developmental cascade of face processing ability could be postulated: face detection → face perception → face memory → face emotion recognition.

According to our latent variable modelling, our postulated cascading model of face processing abilities was confirmed as well-fitting. Thus, it may be suggested that higher levels of face detection facilitate improvements in face perception ability that, in turn, facilitate improvements in face memory ability that, in turn, facilitate improvements in expression recognition ability. Naturally, we are not suggesting that the development of these face processing abilities occurs in an entirely compartmentalised manner. Although it is currently unclear how face processing abilities interact with each other during their development (see Pascalis et al., 2011), one would expect some overlap in development across many of the face processing abilities across time (Weigelt et al., 2014). Nonetheless, our developmental cascading model of face processing is largely consistent with other more long-standing models in the person perception literature. For example, both our cascading model and Bruce and Young’s (1986) model for face recognition emphasises the sequential nature of face processing and the cognitive processes/resources. The proposed cascade model compliments the cognitive perspective of face processing put forth by Bruce and Young (1986) by focusing on the developmental progression of face processing. Furthermore, the neural models (e.g., Gobbini & Haxby, 2007; Haxby et al., 2000) suggest a single representative system for face processing, thus aligning with the perspective of an inter-connected development between face processing abilities as proposed by our cascade model.

One notable difference between the proposed cascading model and these other models is the proposal of more inter-connection between face identity-based abilities and expression recognition. Bruce and Young (1986) proposed two separate systems for processing facial identity and expressions. However, the neurological evidence regarding the separation or convergence for processing facial identity and expressions is inconclusive (see Dalrymple et al., 2017).

Nonetheless, the results associated with the model in Figure 6 suggest that the general trend of development may be consistent with a cascading model. Further research, perhaps especially longitudinal research, is encouraged to help confirm or disconfirm the proposition of our suggested cascading model of face processing abilities.

### Limitations

We acknowledge several limitations associated with our investigation. First, the higher-order intelligence model involving face processing (depicted in Figure 3) aligned with the concept of cognitive load. The strength of associations for each face processing ability increased progressively, starting from face detection (.42) to face memory (.72). Interestingly, expression recognition did not exhibit the highest correlation with the overall face factor, scoring (.60). It is noteworthy that the expression recognition task used in this study encompassed only five basic (prototypical) emotions. While this task did not exhibit a performance ceiling, it likely did not encompass the full spectrum of expressions. For instance, identifying emotions like “annoyed” or “appalled” would likely pose greater difficulty than discerning “angry” or “sad.” Therefore, a more comprehensive expression recognition task encompassing a wider array of subtle expressions might demand more focused attention to distinguish nuanced emotions, potentially tapping into higher levels of crystallised intelligence (*Gc*). With a more refined measure, it is plausible that the loading of expression recognition within the intelligence’s higher-order factor could potentially surpass what was observed within this study.

In addition, there are constraints when applying mediation analysis to entirely cross-sectional data. Criticisms arise because (1) mediation involves a causal process that cannot be fully assessed with data collected at a single time point, and (2) mediation typically entails a longitudinal process (Montoya, 2022). Therefore, the proposed Developmental Cascade Model of Face Processing within this study should be approached cautiously. These models offer insights into potential indirect or causal connections among these variables, supported by some theoretical and empirical evidence. However, a cautious interpretation is essential until further evidence from longitudinal studies or experimental manipulation can confirm the correct temporal sequence of these variables across the human lifetime.

Second, we defined *f* with only four face processing abilities. There are additional facets of face processing ability, for example, long-term familiar face memory (Palermo et al., 2018). Therefore, our face processing ability factor may be considered somewhat incomplete. Furthermore, we measured each face processing ability with only one test. Employing a more comprehensive strategy to specific face processing ability measurement might have yielded stronger results, suggesting our face processing ability findings are likely conservative rather than overstated. A future study with a greater number of face processing tasks (both increasing the dimensions measured and the subtest per dimension) may identify a somewhat larger association between *f* and *g* than that reported in this investigation, as well as between *f* and trait-autism. Furthermore, using only one self-report measure for trait-autism may not be an entirely valid indicator of autistic-like traits. Arguably, an investigation that included a combination of self-ratings and observer-ratings would be advantageous in future research.

General object recognition ability is well-documented to associate with face processing abilities, particularly face memory (Gauthier et al., 2014). In this study, we attempted to control for general object recognition to better estimate the unique effects associated with face processing. However, our measure controlled only for general object recognition specific to the CFMT and not general object detection or perception to match the Mooney Face Test and CFPT’s format. In addition, as our object recognition task used only car images, it likely did not comprehensively account for general object recognition abilities due to the lack of object diversity.

Furthermore, our battery of general cognitive ability subtests was not entirely comprehensive. For example, though the Baddeley Semantic Verification Test would have contributed some Gf variance to the g factor, Gf could have been measured in a superior manner (e.g., matrix reasoning). Ideally, a battery of general cognitive ability would include a minimum of nine Stratum II abilities (Jensen, 1998), each Stratum II ability under *g* (i.e., *Gc, Gs, Gv*, and *Gsm*) would include three to four subtests. The inclusion of three to four subtests for each Stratum II ability would lead to a more valid estimation of those Stratum II abilities, and, therefore, a more valid estimate of the association between *g* and *f*.

We acknowledge that the visual working memory task was associated with relatively poor levels of internal consistency reliability. It was found to correlate significantly and positively with the other cognitive abilities, as expected. Therefore, the lower internal consistency reliability observed for this measure likely did not impact the findings of this investigation appreciably.

Our non-experimental design permitted only estimation of associations between face processing abilities and both autistic-like nonverbal communication and intelligence, precluding any conclusions about causality or effect direction. Our results only suggest that nonverbal communication difficulties might contribute causally to face perception challenges in those with the broader autism phenotype. Specifically, difficulties in autistic-like nonverbal communication may diminish social motivation and hinder the development of face processing abilities, which depend on social exposure to faces (Pascalis et al., 2011) or impair the recognition of essential facial cues necessary for effective face processing. Conversely, it is plausible that deficits in face processing could reduce motivation for social interaction, thereby impairing nonverbal communication abilities (Robertson & Baron-Cohen, 2017). Given our findings, future research should employ training programmes to explore causal effects, particularly by enhancing face processing skills to assess impacts on autistic-like nonverbal communication. Existing evidence supports the efficacy of expression recognition training for autistic individuals (Zhang et al., 2021); thus, this could be a promising focus to investigate the causal relationship between expression recognition and autistic-like nonverbal communication.

## Conclusion

Face processing abilities formed a general face factor (*f*), which was found to be associated positively and appreciably with intelligence (*g*). Thus, increasingly, on both theoretical and empirical grounds, face processing ability is a candidate for inclusion in the CHC model of intelligence, though precisely where it is situated remains to be determined. In addition, the increasingly robust, positive association between intelligence and face memory suggest that developmental prosopagnosia may be clinically operationalised as a learning disability. Finally, autistic-like nonverbal communication ability was found to be associated negatively with face processing abilities, a differential psychology finding that is consistent with the social motivation theory of autism, which supports future research to investigate the potential causal effects between these constructs.

## Supplemental Material

sj-docx-1-qjp-10.1177_17470218251323388 – Supplemental material for The inter-association between face processing, intelligence, and autistic-like nonverbal communication Supplemental material, sj-docx-1-qjp-10.1177_17470218251323388 for The inter-association between face processing, intelligence, and autistic-like nonverbal communication by Dana L Walker, Romina Palermo and Gilles E Gignac in Quarterly Journal of Experimental Psychology

## Notes

1. In addition to identity and emotion, other important information can be discerned from faces, such as trustworthiness, attractiveness, eye gaze, and more. These other face processing abilities are beyond the scope of this study.

2. We note that face memory is related to but not isomorphic with long-term face memory

3. Monk et al. (2022) recommended autistic-preferred terminology in research which is utilised throughout this manuscript.

4. Drop-out rates are unable to be reported due to TestableMinds not providing this data to researchers.

5. We suspect these five cases switched their response strategy during completion of the CFPT, from most similar to least similar (left to right ordering). The substantive results and conclusions of this investigation remained the same including and excluding these five cases.
