# Supplementary material for: The inter-association between face processing, intelligence, and autistic-like nonverbal communication
Source: Q J Exp Psychol (Hove). 2025 Feb 12;78(12):2688–712. doi: 10.1177/17470218251323388 (PMC12638459; doi:10.1177/17470218251323388)
Supplement: sj-docx-1-qjp-10.1177_17470218251323388 – Supplemental material for The inter-association between face processing, intelligence, and autistic-like nonverbal communication [file sj-docx-1-qjp-10.1177_17470218251323388.docx]

Supplementary Material for:

**The Inter-Association Between Face Processing, Intelligence, and Autistic-like Nonverbal Communication**

Dana Walker, Romina Palermo, Gilles G. Gignac

University of Western Australia

**Corresponding author:** Dana Walker

***primary affiliation:***

University of Western Australia - School of Psychological Science

35 Stirling Hwy Crawley

Perth Western Australia 6009

Australia

***secondary affiliation:***

Lingnan University - Department of Psychology

8 Castle Peak Road

Tuen Mun Hong Kong New Territories

Hong Kong

[dana.walker@uwa.edu.au](mailto:dana.walker@uwa.edu.au)

https://orcid.org/0000-0003-2238-5370

**Parcelling Strategy for Latent Variables**

Three item parcels were defined for each latent variable (i.e., face detection, face perception, face memory, general object recognition, expression recognition, crystallised intelligence, processing speed, visuospatial ability, memory span and AQ communication). Specifically, parcel one for each latent variable was comprised of item one and then every third item (i.e., item 4, item 7 etc.), parcel two was comprised of item two and then every third item, and the same strategy was used for parcel 3 (i.e., item three and every third item).

**Figure S1**

*Single-Factor Model for Face Processing Abilities*


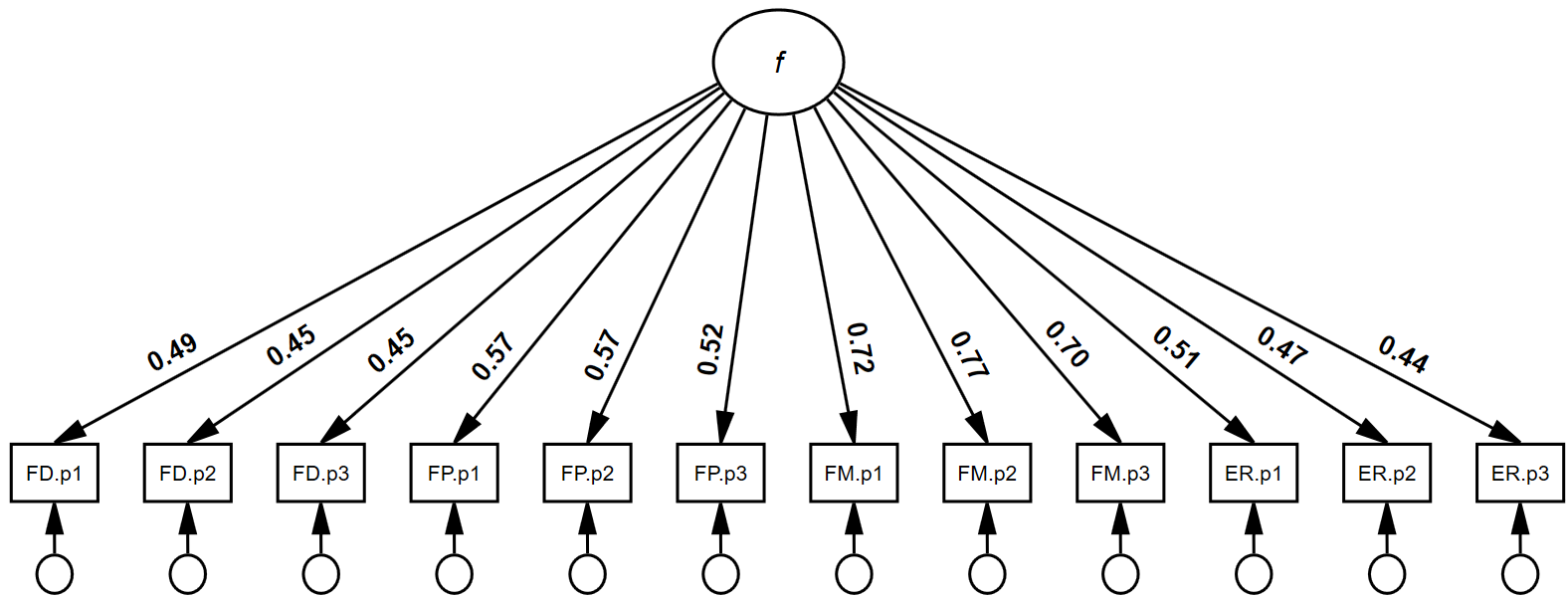


*Note. N* = 253; *f* = general face factor; FD = Face Detection; FP = Face Perception; FM = Face Memory; ER = Expression Recognition; all coefficients were statistically significant, *p* < .05; *χ*^2^(54) = 570.64, *p* < .001, RMSEA = .195, TLI = .485, CFI = .579.

**Figure S2**

*Correlated-Factor Model for Face Processing Abilities*


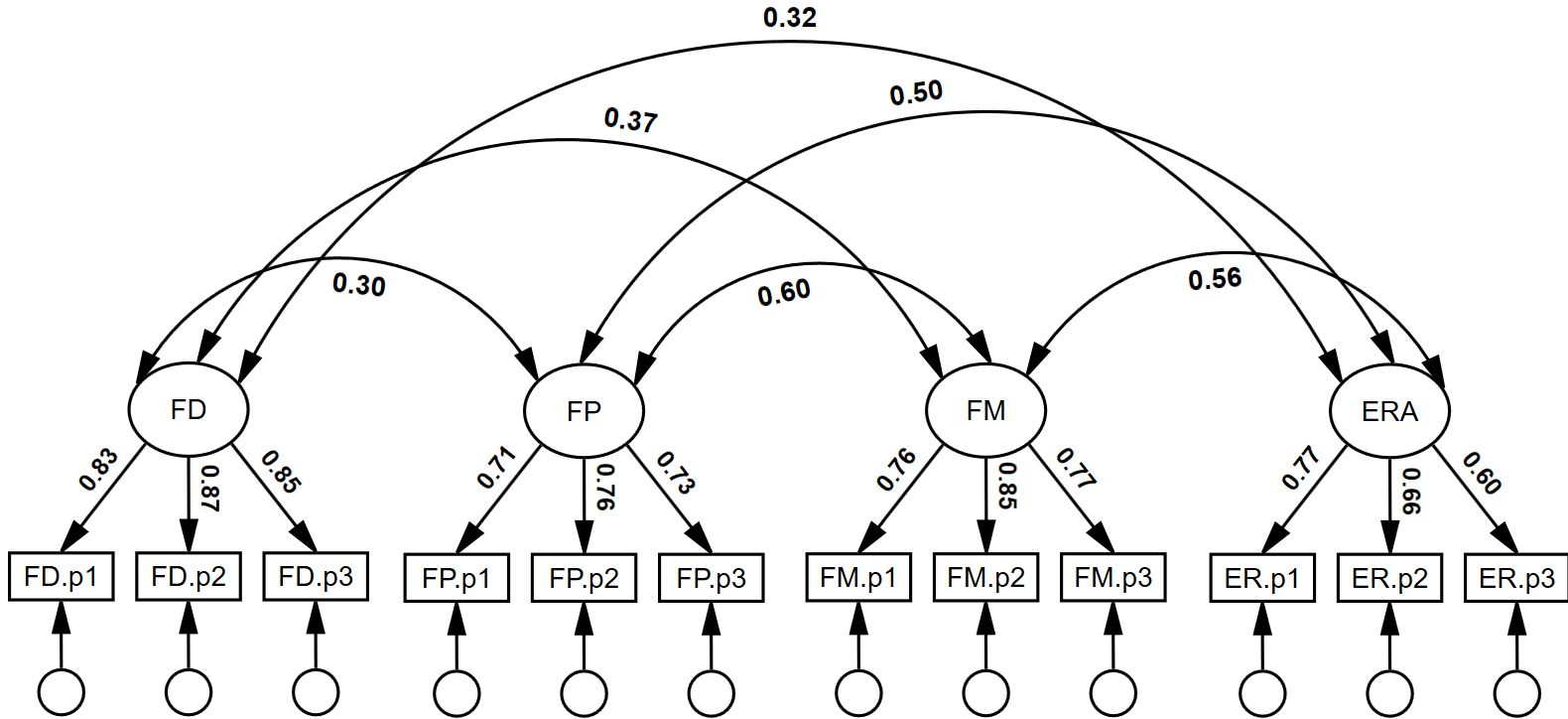


*Note. N* = 253; *f* = general face factor; FD = Face Detection; FP = Face Perception; FM = Face Memory; ER = Expression Recognition; coefficients in bold were statistically significant, *χ*^2^(48) = 46.37, *p* = .540, RMSEA < .001, TLI = 1.002, CFI = 1.000.

**Figure S3**


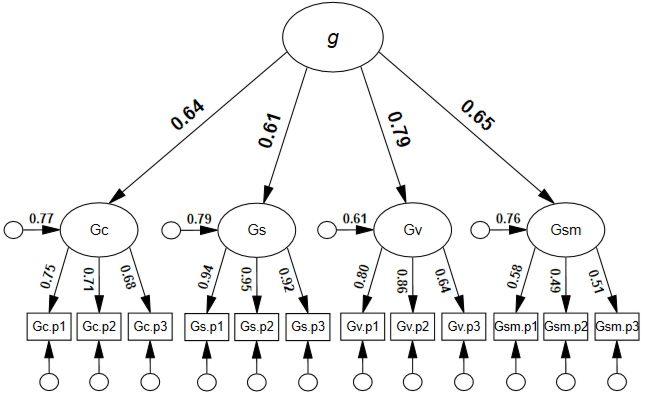
*Higher-Order Four Factor Model for General Intelligence*

*Note. N* = 253; *g* = general cognitive factor; *Gc* = crystallised intelligence; *Gs* = processing speed; *Gv* = visuospatial ability; *Gsm* = memory span; all coefficients were statistically significant, *p* < .05.

All first-order factor residual variances were statistically significant: *Gc*: .77, *p* = .001; *Gs*: .79, *p* = .001; *Gv*: .61, *p* = .003; *Gsm*: .76, *p* = .001; suggesting that each dimension of intelligence was, at least to some degree, unique.

**Figure S4**

*Single-Factor Model for Cognitive Abilities*


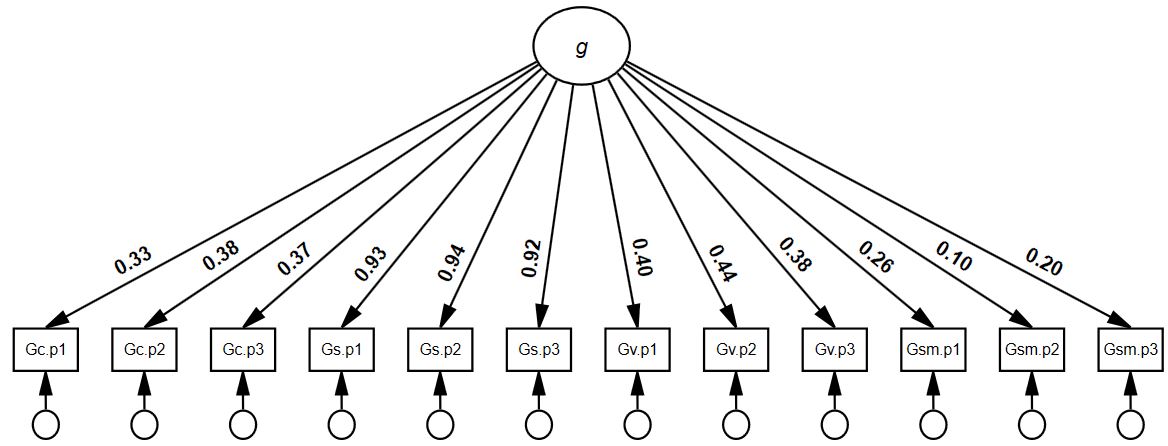


*Note. N* = 253; *g* = general cognitive factor; *Gc* = crystallised intelligence; *Gs* = processing speed; *Gv* = visuospatial ability; *Gsm* = memory span; all coefficients were statistically significant, *p* < .05; model was not well fitting: *χ*^2^(54) = 488.162, *p* < .001, CFI = .700, TLI = .634, RMSEA = .146.

**Figure S5**

*Correlated-Factor Model for Cognitive Abilities*


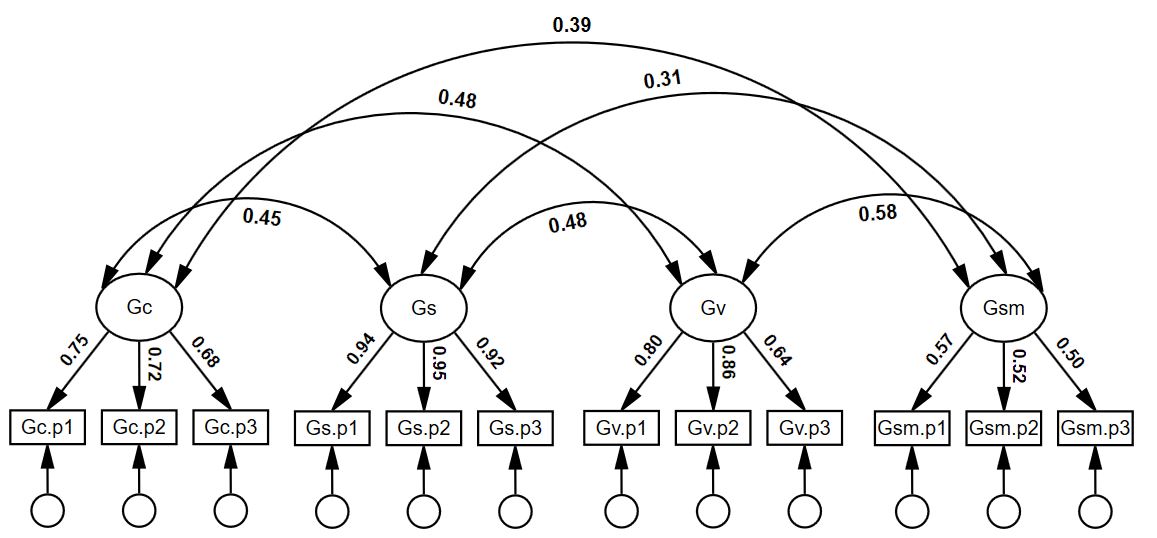


*Note. N* = 253; *g* = general cognitive factor; *Gc* = crystallised intelligence; *Gs* = processing speed; *Gv* = visuospatial ability; *Gsm* = memory span; all coefficients were statistically significant, *p* < .05; model had good close-fit: *χ*^2^(48) = 63.756, *p* = .063, CFI = .989, TLI = .985, RMSEA = .039.

**Figure S6**

*Correlated Model of AQ Dimension with General Intelligence*


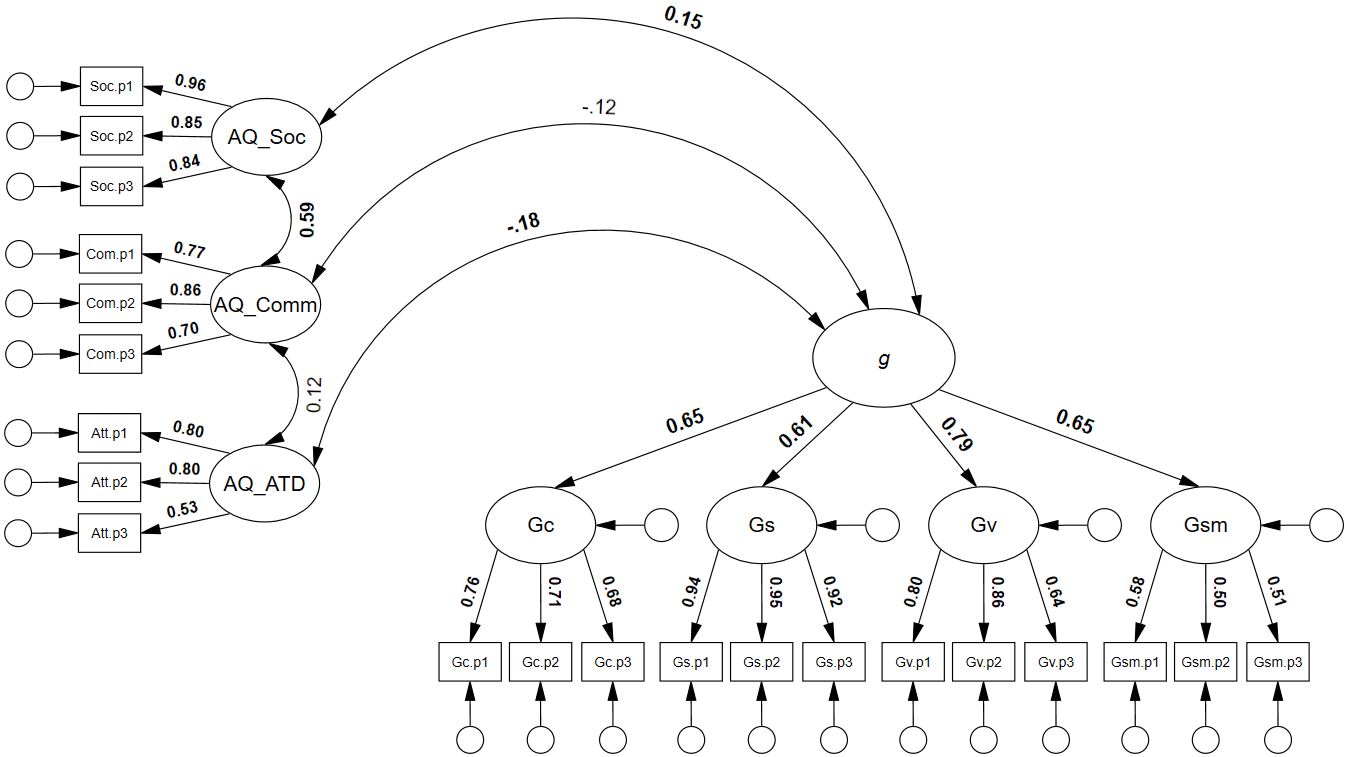


*Note. N* = 253; AQ_Soc = social dimension; AQ_Comm = communication dimension; AQ_ATD = attention to detail dimension; *g* = general cognitive factor; *Gc* = crystallised intelligence; *Gs* = processing speed; *Gv* = visuospatial ability; *Gsm* = memory span; coefficients in bold were statistically significant, *p* < .05; *χ*^2^(181) = 215.852, *p* = .039, CFI = .986, TLI = .984, RMSEA = .052.

**Figure S7**

*
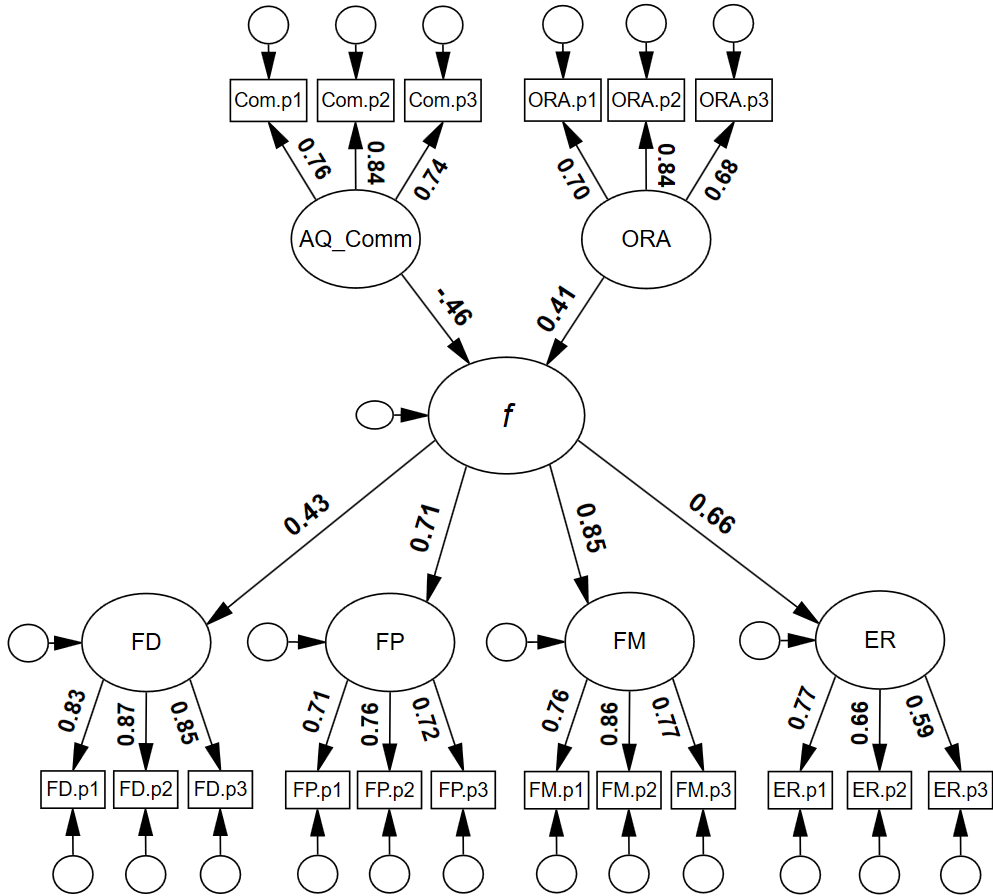
Higher-Order Factor Model for Face Processing with AQ Communication and General Object Recognition*

*Note. N* = 253; AQ_Comm = AQ Communication; ORA = Object Recognition Ability; *f* = general face factor; FD = Face Detection; FP = Face Perception; FM = Face Memory; ER = Expression Recognition; AQ Communication and general object recognition accounted for 38% of the variance in *f* (*R*^2^ = .38, 95%CI = [.24, .51], *p* = .002); all coefficients were statistically significant, *p* < .05.

**Figure S8**

*Structural Equation Model with General Cognitive Factor Predicting the General Face Factor, Controlling for Age*

*
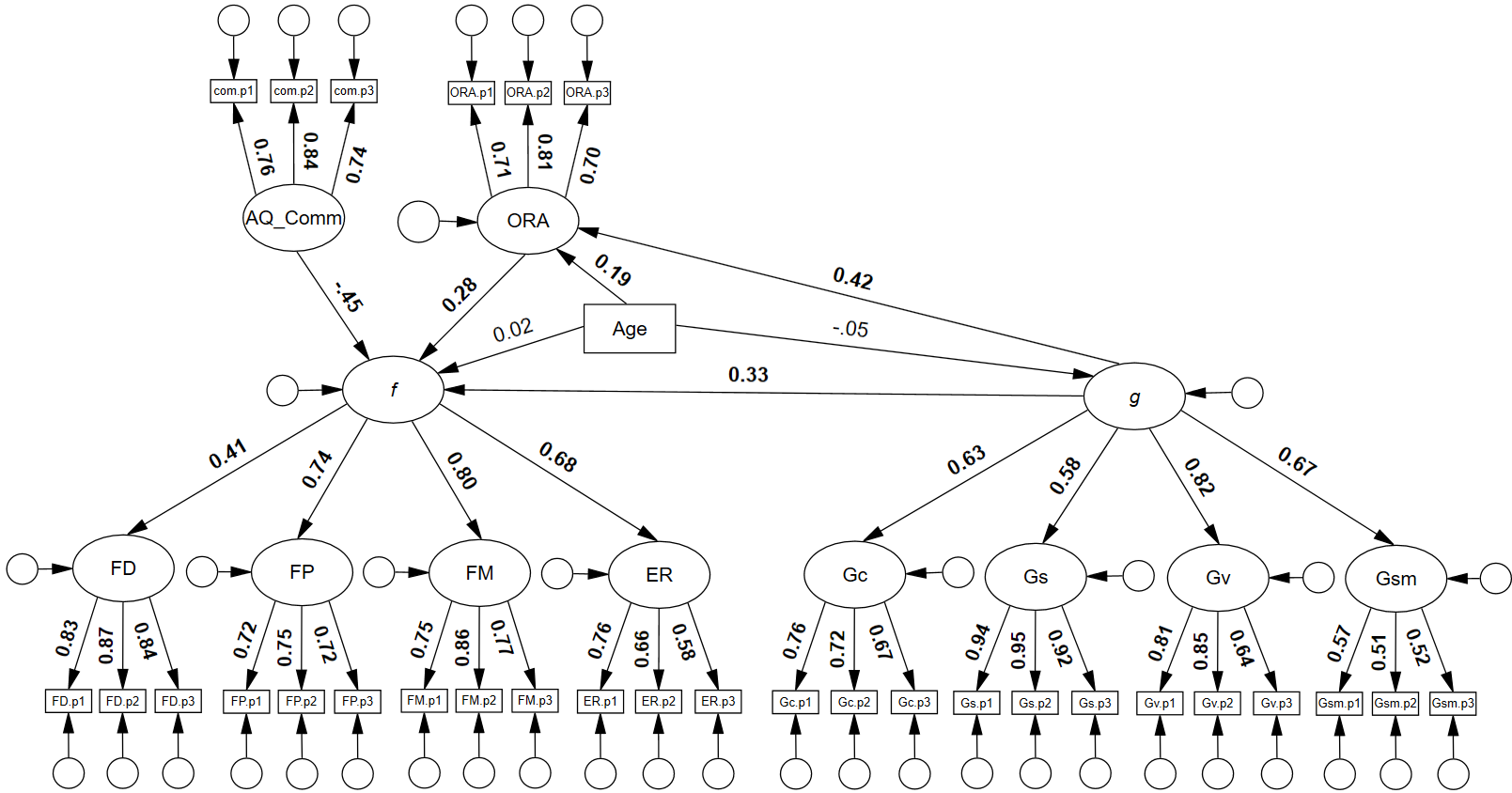
*

*Note. N =* 253; AQ_Comm = AQ Communication; ORA = General Object Recognition; *f* = general face factor; FD = Face Detection; FP = Face Perception; FM = Face Memory; ER = Expression Recognition; *g* = general cognitive factor; *Gc* = crystallised intelligence; *Gs* = processing speed; *Gv* = visuospatial ability; *Gsm* = memory span; coefficients in bold were statistically significant, *p* < .05; *χ*^2^(420) = 531.43, *p* = .001, CFI = .967, TLI = .963, RMSEA = .032.

**Figure S9**


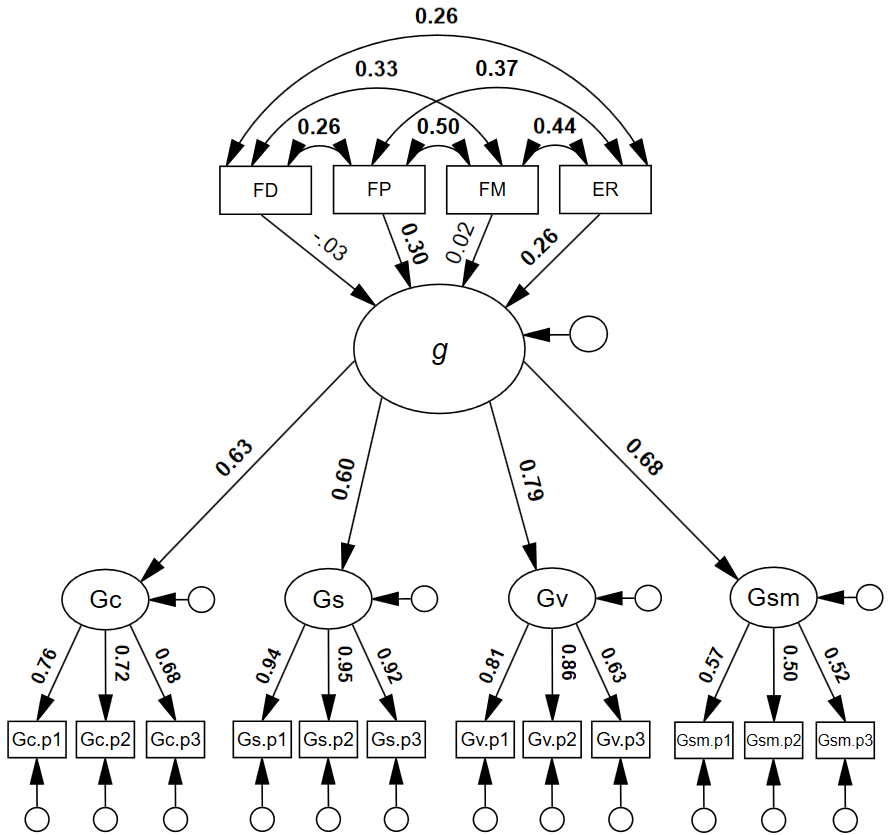
*Higher-Order Model with Face Processing Abilities Predicting General Intelligence*

*Note. N* = 253; *g* = general cognitive factor; *Gc* = crystallised intelligence; *Gs* = processing speed; *Gv* = visuospatial ability; *Gsm* = memory span; coefficients in bold were statistically significant, *p* < .05; The model had good model fit: *χ*^2^(94) = 109.277, *p* = .134, CFI = .991, TLI = .988, RMSEA = .025.
